# Strategies to minimise and monitor biases and imbalances by arm in surgical cluster randomised trials: evidence from ChEETAh, a trial in seven low- and middle-income countries

**DOI:** 10.1186/s13063-022-06852-2

**Published:** 2023-04-05

**Authors:** NIHR Global Research Health Unit on Global Surgery, The Hospital Principle Investigator, Didier Ahogni, Aristide Ahounou, K. Alassan Boukari, Oswald Gbehade, Thierry K. Hessou, Sinama Nindopa, M. J. Bienvenue Nontonwanou, Nafissatou Orou Guessou, Arouna Sambo, Sorekou Victoire Tchati, Affisatou Tchogo, Semevo Romaric Tobome, Parfait Yanto, Isidore Gandaho, Armel Hadonou, Simplice Hinvo, Montcho Adrien Hodonou, Sambo Bio Tamou, Souliath Lawani, Covalic Melic Bokossa Kandokponou, Francis Moise Dossou, Antoine Gaou, Roland Goudou, Marie-Claire Kouroumta, Ismail Lawani, Enrif Malade, Anne Stredy Mkoh Dikao, Joel Nzuwa Nsilu, Pencome Ogouyemi, Marcelin Akpla, Nathan Bisimwa Mitima, Blaise Kovohouande, Cyrille Kpangon, Stephane Laurent Loupeda, Mamonde Victorin Agbangla, Sena Emmanuel Hedefoun, Thierry Mavoha, Juvenal Ngaguene, Janvier Rugendabanga, Rish Romaric Soton, Martin Totin, Mouhamed Agbadebo, Irene Akpo, Hubert Dewamon, Martin Djeto, Aissatou Hada, Monsede Hollo, Albert Houndji, Anasthasie Houndote, Sylvestre Hounsa, Expedit Kpatchassou, Hugues Yome, Mohamed Moussa Alidou, Eric Jerry Bara, B. T. Bonheur Dossou Yovo, Robert Guinnou, Souleymane Hamadou, H. Pauline Kola, Nabil Moussa, Boniface Cakpo, Lolyta Etchisse, Emery Hatangimana, Moise Muhindo, Katia Sanni, Agossou Barthelemy Yevide, Hermann Agossou, Fiston Basirwa Musengo, Hulrich Behanzin, Djifid Morel Seto, Bill Armstrong Alia, Arnaud Alitonou, Y. Edith Mehounou, Lucien Agbanda, Julien Attinon, Marcel Gbassi, Nounagnon Rene Hounsou, Regina Acquah, Charles Banka, Derick Esssien, Romeo Hussey, Yakubu Mustapha, Kojo Nunoo-Ghartey, Grace Yeboah, Luke A. Aniakwo, Margarey N. M. Adjei, Yvonne Adofo-Asamoah, Meshach M. Agyapong, Thomas Agyen, Baba A. B. Alhassan, Mabel P. Amoako-Boateng, Anthony Baffour Appiah, Josephine Ashong, Joseph K. Awindaogo, Benjamin B. Brimpong, Makafui S. C. J. K. Dayie, Donald Enti, Wendy W. Ghansah, Jude E. Gyamfi, Patience Koggoh, Richard Kpankpari, Vincent Kudoh, Samuel Mensah, Philip Mensah, Isabella N. Morkor Opandoh, Martin T. Morna, Michael Nortey, Emelia Odame, Emmanuel O. Ofori, Sandra Quaicoo, Elizabert M. Quartson, Cynthia Teye-Topey, Makafui Yigah, Safia Yussif, Esther Adjei-Acquah, Vera O. Agyekum-Gyimah, Eric Agyemang, Arko Akoto-Ampaw, Forster Amponsah-Manu, Temitope E. Arkorful, Moses A. Dokurugu, Nanabanyin Essel, Aja Ijeoma, Emmanuel L. Obiri, Richard Ofosu-Akromah, Karen N. D. Quarchey, Leslie Adam-Zakariah, Aaron B. Andoh, Esther Asabre, Ruby A. Boateng, Barbara Koomson, Atta Kusiwaa, Adeline Naah, Ato Oppon-Acquah, Benjamin A. Oppong, Emma A. Agbowada, Ameley Akosua, Ralph Armah, Christopher Asare, Lawrence K. B. Awere-Kyere, Amanda Bruce-Adjei, Nana Ama Christian, Delali A. Gakpetor, Korankye K. Kennedy, Jacqueline Mends-Odro, Ambe Obbeng, Doris Ofosuhene, Dorcas Osei-Poku, Zelda Robertson, Dorcas O. Acheampong, Jane Acquaye, Juliana Appiah, Joshua Arthur, Jonathan Boakye-Yiadom, Anita Eseenam Agbeko, Frank E. Gyamfi, Bertina B. Nyadu, Samira Abdulai, Nii A. Adu-Aryee, Nelson Agboadoh, Erica Akoto, Joachim K. Amoako, Nicholas T. Aperkor, Wilfred K. Asman, Godsway S. Attepor, Antoinette A. Bediako-Bowan, Kwaku Boakye-Yiadom, George D. Brown, Florence Dedey, Victor K. Etwire, Benjamin S. Fenu, Philemon K. Kumassah, Linda A. Larbi-Siaw, Josephine Nsaful, David O. Olatola, Sandra E. Tsatsu, Theodore Wordui, Iddrisu I. A. Abdul-Aziz, Fatao Abubakari, Johnson Akunyam, Gilbert A. G. Anasara, Cletus Ballu, Charles G. Barimah, Guy C. Boateng, Ponala W. Kwabena, Seidu M. Kwarteng, Prosper T. Luri, Kennedy Ngaaso, David K. D. Ogudi, Vivian Adobea, Amos Bennin, Stanley Doe, Ruth Sarfo Kantanka, Ephraim Kobby, Collins Kyeremeh, Edwin Osei, Prince Yeboah Owusu, Frank Owusu, Clement Sie-Broni, Marshall Zume, Saba Abdul-Hafiz, Daniel K. Acquah, Shamsudeen M. Adams, Mohammed S. Alhassan, Munira Amadu, Samuel A. Asirifi, Martin Awe, Millicent Azanlerigu, Mathias K. Dery, Yenli Edwin, Abantanga Atindaana Francis, Gbana Limann, Aloysius Maalekuu, Hawa Malechi, Sheriff Mohammed, Ibrahim Mohammed, Kareem Mumuni, Bernard A. Ofori, Jonathan I. K. Quansah, Anwar S. Seidu, Stephen Tabiri, Shekira Yahaya, Emmanuel Kojo Acquah, Jaabir Alhassan, Percy Boakye, Christian L. Coompson, Addo K. Gyambibi, Ametepe Jeffery-Felix, Bismark E. Kontor, Ruth Manu, Elijah Mensah, Gifty Naah, Carmen Noufuentes, Abraham Sakyi, Ramkaran Chaudhary, Sanjeev Misra, Puneet Pareek, Manish Pathak, Dharma R. Poonia, Kirti K. Rathod, Mahaveer S. Rodha, Naveen Sharma, Nivedita Sharma, Subhash C. Soni, Vaibhav K. Varsheney, Jeevan R. Vishnoi, Deepak K. Garnaik, Farhanul Huda, Manoj J. Lokavarapu, Neha Mishra, Rohit Ranjan, Rajkumar K. Seenivasagam, Shanky Singh, Pratik Solanki, Raunak Verma, Enono Yhoshu, Suzan John, Jeffery A. Kalyanapu, Ananta Kutma, Sanish Philips, Arun K. Gautham, Alice Hepzibah, Grace Mary, Deepak S. Singh, Eunice S. Abraham, Chetana Chetana, Amos Dasari, Prashant Dummala, Chinta S. Gold, Jurgen Jacob, Jeremiah N. Joseph, Elizabeth N. Kurien, Priya Mary, Arpit J. Mathew, Amy E. Mathew, Danita D. Prakash, Oliver Samuel, Ashwin Sukumar, Niyah Syam, Rose Varghese, Alisha Bhatt, William Bhatti, Tapasya Dhar, Dhruva N. Ghosh, Ankush Goyal, Sunita Goyal, Monika A. Hans, Parvez D. Haque, Deepak Jain, Rita Jain, Jyoti Jyoti, Savleen Kaur, Karan Kumar, Anil Luther, Amit Mahajan, Kavita Mandrelle, Vishal Michael, Partho Mukherjee, Reuben Rajappa, Vivin Daniel Sam, Prashant Singh, Atul Suroy, Ravinder Singh Thind, Sreejith K. Veetil, Rahul Williams, D. Sreekar, Esther R. Daniel, Smitha E. Jacob, Mark R. Jesudason, Pushplatha Kumari, Rohin Mittal, Soosan Prasad, Vasanth Mark Samuel, Bharat Shankar, Srujan Sharma, Moonish V. Sivakumar, Suraj Surendran, Anita Thomas, Paul Trinity, Sudheer Kanchodu, K. Leshiini, Sundeep S. Saluja, Ashok K. Attri, Ishan Bansal, Sanjay Gupta, Monika Gureh, Simran Kapoor, Manisha Aggarwal, Vinoth Kanna, Harmanjot Kaur, Ashwani Kumar, Simrandeep Singh, Gurtaj Singh, Viju John, Mohammed Adnan, Nivesh Agrawal, Uttkarsh Kumar, Pardeep Kumar, S. Abhishek, Vikram Sehrawat, Deepak Singla, Gaurav Thami, Vijay Kumar, Stanley Mathew, Murlidhar V. Pai, P. S. Prabhu, P. T. Sundeep, Naseem Akhtar, Arun Chaturvedi, Sameer Gupta, Vijay Kumar, Puneet Prakash, Shiv Rajan, Mohit Singh, Abhilasha Tripathi, Philip V. Alexander, Josy Thomas, Pradeep Zechariah, Vijay A. Ismavel, Moloti Kichu, Carolin V. Solomi, Rahul A. Alpheus, Ashish Victor Choudhrie, Rashmi Jacob Gunny, Susan Joseph, Muneer A. Malik, Nitin J. Peters, Neha Pundir, Ram Samujh, Hafsa I. Ahmed, Gowhar Aziz, Nisar A. Chowdri, Rayees A. Dar, Robindera Kour, Imtiyaz Mantoo, Asif Mehraj, Fazl Q. Parray, Najmus Saqib, Zamir A. Shah, Rauf A. Wani, Subrat Raul, Komal Rautela, Rajeev Sharma, Nishu Singh, Rakesh Vakil, Priyanka Chowdhury, Sona Chowdhury, Sonia Mathai, Pragyanmai Nayak, Bipradas Roy, Andrea S. Alvarez Villaseñor, Kriscia V. Ascencio Díaz, Victor J. Avalos Herrera, Francisco J. Barbosa Camacho, Aldo Bernal Hernández, Elyoenai Bonilla Ahumada, Irma V. Brancaccio Pérez, Miguel A. Calderón Llamas, Guadalupe Castillo Cardiel, Guillermo A. Cervantes Cardona, Gabino Cervantes Guevara, Enrique Cervantes Perez, Maria Chávez, Jonathan M. Chejfec Ciociano, Luis R. Cifuentes Andrade, Ana O. Cortés Flores, Edgar J. Cortes Torres, Tania A. Cueto Valadez, Andrea E. Cueto Valadez, Esteban Cueva Martinez, Paulina Domínguez Barradas, Isaac Esparza Estrada, Paola Flores Becerril, Jose A. Flores Cardoza, Clotilde Fuentes Orozco, Luis A. García González, Benjamín García Reyna, Eduardo Gómez Sánchez, Jaime L. González Bojorquez, Eduardo González Espinoza, Alejandro González Ojeda, Fanny Y. González Ponce, Cristhian S. Guerrero Ramírez, José A. Guzmán Barba, Bertha G. Guzmán Ramírez, Mario J. Guzmán Ruvalcaba, Daniel A. Hérnandez Alva, Silvia A. Ibarra Camargo, Juan C. Ibarrola Peña, Martin Islas Torres, Jorge Jiménez Tornero, Zayra M. Lara Pérez, Roberto Mares País, Mel P. Mellado Tellez, Roberto C. Miranda Ackerman, Damián Mora Santana, Gilberto Morgan Villela, Rodrigo Nájar Hinojosa, Cesar Nuño Escobar, Itzel Ochoa Rodríguez, Oscar Olvera Flores, Angelica Ortega Barreiro, Jacqueline Osuna Rubio, Luis R. Pacheco Vallejo, Víctor H. Pérez Bocanegra, Jose V. Pérez Navarro, Francisco J. Plascencia Posada, María A. Quirarte Hernández, Luis R. Ramirez Gonzalez, Emilio A. Reyes Elizalde, Evelia V. Romo Ascencio, Cornelio Ruelas Bravo, Carlos B. Ruiz Velasco, José A. Sánchez Martínez, Guillermo Sanchez Villaseñor, José I. Sandoval Pulido, Alejandro G. Serrano García, Luis O. Suárez Carreón, Juan J. Tijerina Ávila, Jesus O. Vega Gastelum, Melissa L. Vicencio Ramirez, Maria F. Zarate Casas, Carlos J. Zuloaga Fernández del Valle, Jesus Antonion Aguilar Mata, Miguel Antonio Calderon Vanegas, Rocio Guadalupe Cano Arias, Carlos Colunga Tinajero, Fernanda Diaz Samano, Fernando Duque Zepeda, Brenda Vanessa Enriquez Barajas, Gerardo Gallardo Banuelos, Marijose De Cristo Gonzalez Calvillo, Francisco Ibanez Ortiz, Maryzela Lazo Ramirez, Gerardo Lopez Arroyo, Laura Olivia Montano Angeles, David Giovanny I. Morales Iriarte, Angelo Fernando Mortola Lomeli, Jose Esteban Orozco Navarro, Jaime Orozco Perez, Damaris Orozco Ramirez, Laura Gabriela Pena Baolboa, Jesus Pizarro Lozano, Guillermo Yanowsky Reyes, Monica N. Castillo, Ana Camille G. Dominguez, Dorihela H. Mellado, Jesus Flavio M. Morales, Luz del Carmen H Namur, Jose Alberto A. Pesquera, Laura Martinez Perez Maldonado, Antonio Ramos De la Medina, Katya Bozada-Gutierrez, Ana Florencia Casado-Zarate, Roberto Delano-Alonso, Jose Herrera-Esquivel, Mucio Moreno-Portillo, Mario Trejo-Avila, Roland Kevin Cethorth Fonseca, Edgard Efren Lozada Hernandez, Bruno Crocco Quiros, Jairo Arturo Rodriguez Ramirez, Gabriela Ambriz-González, Mitzi R. Becerra Moscoso, Ishtar Cabrera-Lozano, Ana B. Calderón-Alvarado, Francisco J. León-Frutos, Erick E. Villanueva-Martínez, Aisha Abdullahi, Maimuna Abubakar, Mohammed S. Aliyu, Mudi Awaisu, Fadimatu Bakari, Abigail Olajumoke Balogun, Mohammed Bashir, Ahmad Bello, Muhammad Daniyan, Kehinde Michael Duromola, Stephen G. Gana, Mukoro Duke George, Justina Gimba, Isaac Gundu, Lambert Onahi Iji, Aminat O. Jimoh, Afolabi K. Koledade, Ahmad T. Lawal, Bilkisu K Lawal, Aisha Mustapha, Stanley Emeka Nwabuoku, Oluseyi O. Ogunsua, Ifeanyi Fidelis Okafor, Ethos Ike Okorie, Nasir Oyelowo, Ibrahim A. Saidu, Tunde T. Sholadoye, Ibrahim Sufyan, Musliu Adetola Tolani, Aliyu Muhammad Tukur, Ahmad Shehu Umar, Aminatu M. Umar, Hajara Umaru-Sule, Mohammed Usman, Anisah Yahya, Alfa Yakubu, Salisu Abeku Yusuf, Abdulhafiz A. Abdulkarim, Lawal Barau Abdullahi, Muzzammil Abdullahi, Khadija A. Ado, Nura U. Aliyu, Lofty-John Chukwuemeka Anyanwu, Sulaiman M. Daneji, Mahmoud Kawu Magashi, Mohammad A. Mohammad, Abubakar Bala Muhammad, Saminu S. Muhammad, Bello Abodunde Muideen, Calistus U. Nwachukwu, Suleiman B. Sallau, Abdulrahman A. Sheshe, Abdulmajeed Soladoye, Idris Usman Takai, Garzali I. Umar, Abubakar Yahaya, Lubabatu Abdulrasheed, Joel A. Adze, Lydia R. Airede, Bashiru Aminu, Stephen B. Bature, Firdaws Bello-Tukur, Damai Chinyio, Sharon A. N. Duniya, Moses C. Galadima, Babatunde K. Hamza, Samaila Joshua, Stephen A. Kache, Williams Y. Kagomi, Ifeanyi A. Kene, Jamila Lawal, Jerry G. Makama, Caleb Mohammed, Amina A. Mohammed-Durosinlorun, Deborah Nuwam, Danjuma Sale, Abdulrasheed Sani, Salome Tabara, Mathew C. Taingson, Emmanuel Usam, Josiah Yakubu, Folasade Adegoke, Oluwasuyi Ige, Tunde A. Odunafolabi, Chukwuma E. Okereke, Oluwafemi O. Oladele, Oluwaseun H. Olaleye, Oyetunde O. Olubayo, Olukayode P. Abiola, Henry O. Abiyere, Idowu O. Adebara, Gbadebo T. C. Adeleye, Adebayo A. Adeniyi, Olumide E. Adewara, Olabisi T. Adeyemo, Ademola A. Adeyeye, Abimbola L. Ariyibi, Babatunde S. Awoyinka, Olumide M. Ayankunle, Olakunle F. Babalola, Adewumi Bakare, Tajudeen I. B. Bakare, Oluseyi O. Banjo, Peter A. Egharevba, Oluwafemi S. Fatudimu, John A. Obateru, Oluremi J. Odesanya, Owolabi D. Ojo, Abiodun I. Okunlola, Cecilia K. Okunlola, Adewale T. Olajide, Tesleem O. Orewole, Adedayo I. Salawu, Moruf A. Abdulsalam, Aderinsola T. Adelaja, Olalekan T. Ajai, Olukemi Akande, Noble Anyanwu, Kazeem M. Atobatele, Oludayo Oluwaseyi Bakare, Grace Eke, Omolara M. Faboya, Zainab O. Imam, Francisca C. Nwaenyi, Ayokunle A. Ogunyemi, Mobolaji A. Oludara, Olufunmilade A. Omisanjo, Chinonso U. Onyeka, Olabode A. Oshodi, Yusuf A. Oshodi, Yemisi Oyewole, Omotade S. Salami, Omolara M. Williams, Esther Abunimye, Adesoji O. Ademuyiwa, Adebunmi Adeoluwa, Adedotun Adesiyakan, Victoria Ibukunoluwa Adeyeye, Moses Vincent Agbulu, Opeyemi Rebecca Akinajo, David O. Akinboyewa, Felix M. Alakaloko, Iyabo O. Alasi, Michael Amao, Christiana Ashley-Osuzoka, Oluwole A. Atoyebi, Olanrewaju S. Balogun, Christopher O. Bode, Maryam Oluwatobi Busari, Nnamdi Jonathan Duru, Glory Bassey Edet, Olumide A. Elebute, Francis Chinonso Ezenwankwo, Adedeji L. Fatuga, Christianah Gbenga-Oke, George C. Ihediwa, Emmanuel Sylvester Inyang, Adesola I. Jimoh, Jubril Oladayo Kuku, Oluwaseun A. Ladipo-Ajayi, Abdulrazzaq O. Lawal, Ayomide Makanjuola, Christian Chigoze Makwe, Chinelo Victoria Mgbemena, Samuel U. Nwokocha, Moses Adebisi Ogunjimi, Ephraim Okwudiri Ohazurike, Rufus W. Ojewola, Moyosoluwa Eunice Badedale, Chike J. Okeke, Adeyemi A. Okunowo, Abraham T. Oladimeji, Thomas O. Olajide, Olabisi Olanrewaju, Olawunmi Olayioye, Oluwaseun O. Oluseye, Stephen Olutola, Kenneth Onyekachi, Adeola Ayoola Orowale, Emili Osariemen, Adedapo Olumide Osinowo, Benedetto Osunwusi, Emmanuel Owie, Christianah Bidemi Oyegbola, Justina O. Seyi-Olajide, Adaiah P. Soibi-Harry, Manuella Talla Timo, Aloy Okechukwu Ugwu, Emmanuel Ojo Williams, Innocent O. Duruewuru, Ochonma A. Egwuonwu, Okechukwu Hyginus Ekwunife, James J. Emeka, Victor Ifeanyichukwu Modekwe, Chimdiebele Daisy Nwosu, Sylvester O. Obiechina, Ahuizechukwu E. Obiesie, Celestine I. Okafor, Theophilus O. Okonoboh, Chukwuemeka Okoro, Odili A. Okoye, Onyekachi A. Onu, Chukwudubem C. Onyejiaka, Chisom Faith Uche, Joseph O. Ugboajah, Jideofor Okechukwu Ugwu, Kenneth Ugwuanyi, Chuka Ugwunne, Akeem A. Adeleke, Akinfolarin C. Adepiti, Adewale A. Aderounmu, Abdulhafiz O. Adesunkanmi, Adewale O. Adisa, Samuel C. Ajekwu, Olusegun K. Ajenifuja, Olusegun I. Alatise, Tajudeen A. Badmus, Tajudeen O. Mohammed, Olalekan Olasehinde, Abdulkadir A. Salako, Oludayo A. Sowande, Ademola O. Talabi, Funmilola O. Wuraola, Paul Aderemi Adegoke, Abidemi Akinloye, Ayodeji Akinniyi, Joseph Ejimogu, Ideyonbe Samuel Eseile, Olakayode Olaolu Ogundoyin, Amos Okedare, Dare Isaac Olulana, Omolara Omotola, Francis Sanwo, Collins C. Adumah, Adewale O. Ajagbe, Olugbenga P. Akintunde, Opeyemi Q. Asafa, Kehinde Awodele, Amogu K. Eziyi, Adeniyi O. Fasanu, Olufemi O. Ojewuyi, Abiodun R. Ojewuyi, Abisola E. Oyedele, Oluwaseun A. Taiwo, Habiba I. Abdullahi, Nathaniel D. Adewole, Teddy E. Agida, Eunice E. Ailunia, Oseremen Aisuodionoe-Shadrach, Godwin O. Akaba, Janet Alfred, Terkaa Atim, Kehinde G. Bawa, John Y. Chinda, Esther B. Daluk, Sefiu B. Eniola, Augusta O. Ezenwa, Stephen E. Garba, Ndubuisi Mbajiekwe, Philip M. Mshelbwala, Ngozi O. Ndukwe, Idoko P. Ogolekwu, Alexander A. Ohemu, Samson Olori, Olabisi O. Osagie, Samuel A. Sani, Salisu Suleiman, Helen Sunday, Nancy O. Tabuanu, Aminu M. Umar, Peter I. Agbonrofo, Alexander I. Arekhandia, Morrison E. Edena, Raymond A. Eghonghon, Joel E. Enaholo, Genesis Ida, Stanley N. Ideh, Oseihie I. Iribhogbe, Omorodion O. Irowa, Maradona E. Isikhuemen, Oluwatomi R. Odutola, Kester O. Okoduwa, Scott O. Omorogbe, David Oruade, Osasumwen T. Osagie, Osarenkhoe Osemwegie, Rukiyat A. Abdus-Salam, Sikiru Adekola Adebayo, Oluwasanmi A. Ajagbe, Akinlabi E. Ajao, Gboyega Ajibola, Omobolaji O. Ayandipo, Kelvin I. Egbuchulem, Hyginus O. Ekwuazi, Peter Elemile, Adegbolahan Fakoya, Oluwasegun C. Idowu, David O. Irabor, Taiwo A. Lawal, Olatunji O. Lawal, Olakayode O. Ogundoyin, Oluwabukade Ojediran, Naomi Olagunju, Akinsola T. Sanusi, Augustine O. Takure, Lukman Olajide Abdur-Rahman, Mary Oluwadamilola Adebisi, Nurudeen Abiola Adeleke, Rafiat Tinuola Afolabi, Isiaka Ishola Aremu, Jibril Oyekunle Bello, Robiat Bello, Abdulwahab Lawal, Saheed Abolade Lawal, Adeolu Ojajuni, Sabur Oyewale, Hadijat Olaide Raji, Olayinka Sayomi, Asimiyu Shittu, Victor Abhulimen, Patrick O. Igwe, Ikechukwu Enyinnaya Iweha, Raphael E. John, Nnyonno Okoi, Philemon E. Okoro, Vaduneme Kingsley Oriji, Ibiene T. Oweredaba, Japhet Mizero, Immaculee Mutimamwiza, Francoise Nirere, Irenee Niyongombwa, Jean Paul Majyabere, Anastase Byaruhanga, Rongin Dukuzimana, Jean Aimable Habiyakare, Marie Gloriose Nabada, Marcel Uwizeye, Mathias Ruhosha, Joselyne Igiraneza, Faustine Ingabire, Aloys Karekezi, Jean Pierre Masengesho, Christophe Mpirimbanyi, Lydia Mukamazera, Clemence Mukangabo, Jean Paul Niyomuremyi, Gabriel Ntwari, Celestin Seneza, Divine Umuhoza, Sosthene Habumuremyi, Alphonsine Imanishimwe, Salathiel Kanyarukiko, Francine Mukaneza, Deborah Mukantibaziyaremye, Aphrodis Munyaneza, Gibert Ndegamiye, Pierrine Nyirangeri, Ronald Tubasiime, Jean Claude Uwimana, Moses Dusabe, Emelyne Izabiriza, Hope Lydia Maniraguha, Christophe Mpirimbanyi, Josiane Mutuyimana, Olivier Mwenedata, Elisee Rwagahirima, Job Zirikana, Isaie Sibomana, Desire Rubanguka, Josine Umuhoza, Roda Uwayezu, Leoncie Uzikwambara, Aime Dieudonne Hirwa, Elysee Kabanda, Salomee Mbonimpaye, Christine Mukakomite, Piolette Muroruhirwe, Herbert Butana, Moise Dusabeyezu, Athanasie Mukasine, Jean N. Utumatwishima, Mediatrice Batangana, Georges Bucyibaruta, Sosthene Habumuremyi, Jean de Dieu Haragirimana, Alphonsine Imanishimwe, Allen J. C. Ingabire, Violette Mukanyange, Emmanuel Munyaneza, Emmanuel Mutabazi, Espoir Mwungura, Isaie Ncogoza, Faustin Ntirenganya, Jeannette Nyirahabimana, Dancilla Nyirasebura, Christian Jean Urimubabo, Anaclet Dusabimana, Sam Kanyesigye, Robert Munyaneza, Jean Yves Shyirakera, Maria Fourtounas, Mary Augusta Adams, Chikwendu Jeffrey Ede, Gabriella Hyman, Mpho Nosipho Mathe, Rachel Moore, Ncamsile Anthea Nhlabathi, Hlengiwe Samkelisiwe Nxumalo, Nnosa Sentholang, Mmule Evelyn Sethoana, Paul Wondoh, Zain Ally, Aimee Domingo, Philip Munda, Chido Nyatsambo, Victor Ojo, Rudo Pswarayi

**Affiliations:** NIHR Global Health Research Unit on Global Surgery, Institute of Applied Health Research, University of Birmingham, B15 2TH Birmingham, UK

**Keywords:** Surgical site infection, Abdominal surgery, Global surgery, Global health, Cluster randomised controlled trial, Bias, Research methodology, Quality assurance, Trial management

## Abstract

**Background:**

Cluster randomised controlled trials (cRCT) present challenges regarding risks of bias and chance imbalances by arm. This paper reports strategies to minimise and monitor biases and imbalances in the ChEETAh cRCT.

**Methods:**

ChEETAh was an international cRCT (hospitals as clusters) evaluating whether changing sterile gloves and instruments prior to abdominal wound closure reduces surgical site infection at 30 days postoperative. ChEETAh planned to recruit 12,800 consecutive patients from 64 hospitals in seven low-middle income countries. Eight strategies to minimise and monitor bias were pre-specified: (1) minimum of 4 hospitals per country; (2) pre-randomisation identification of units of exposure (operating theatres, lists, teams or sessions) within clusters; (3) minimisation of randomisation by country and hospital type; (4) site training delivered *after* randomisation; (5) dedicated ‘warm-up week’ to train teams; (6) trial specific sticker and patient register to monitor consecutive patient identification; (7) monitoring characteristics of patients and units of exposure; and (8) low-burden outcome-assessment.

**Results:**

This analysis includes 10,686 patients from 70 clusters. The results aligned to the eight strategies were (1) 6 out of 7 countries included ≥ 4 hospitals; (2) 87.1% (61/70) of hospitals maintained their planned operating theatres (82% [27/33] and 92% [34/37] in the intervention and control arms); (3) minimisation maintained balance of key factors in both arms; (4) post-randomisation training was conducted for all hospitals; (5) the ‘warm-up week’ was conducted at all sites, and feedback used to refine processes; (6) the sticker and trial register were maintained, with an overall inclusion of 98.1% (10,686/10,894) of eligible patients; (7) monitoring allowed swift identification of problems in patient inclusion and key patient characteristics were reported: malignancy (20.3% intervention vs 12.6% control), midline incisions (68.4% vs 58.9%) and elective surgery (52.4% vs 42.6%); and (8) 0.4% (41/9187) of patients refused consent for outcome assessment.

**Conclusion:**

cRCTs in surgery have several potential sources of bias that include varying units of exposure and the need for consecutive inclusion of all eligible patients across complex settings. We report a system that monitored and minimised the risks of bias and imbalances by arm, with important lessons for future cRCTs within hospitals.

**Supplementary Information:**

The online version contains supplementary material available at 10.1186/s13063-022-06852-2.

## Introduction

Surgical innovations often involve complex, across-team interventions that require behavioural change [1–3]. Evaluation of these innovations in a randomised trial requires a cluster randomised design because of the high risk of contamination between intervention and control arms with individual patient randomisation, as well as the logistical and practical issues around the delivery of the intervention [4–6]. However, few cluster randomised controlled trials (cRCTs) have been conducted to date in surgery [7]. As a result, methods for high-quality delivery of cRCTs in surgical settings are still evolving [8, 9].

A major methodological challenge in cRCTs is minimising bias and arm imbalance. In cluster randomisation, chance imbalances in patient characteristics are likely to occur [10]. This can lead to a risk confounding bias. Selection bias occurs where there is incomplete identification and recruitment of eligible patients within a cluster overall. This can lead to a sample that is unrepresentative of the target population compromising external validity and/or an unfair comparison of the trial arms due to the differences in known and unknown confounders compromising internal validity. In addition, the lack of allocation concealment can also impact cluster size variability resulting from selection bias, causing further imbalances.

In surgical trials with cluster randomisation, there are specific challenges. The risk of bias is raised when it is necessary to unmask the clusters to their randomised group prior to the recruitment of eligible participants (i.e. where allocation concealment is not possible). In the context of a surgical cRCT, recruitment of participants occurs dynamically over a period of time and in different settings (e.g. preoperative clinic, intraoperatively in theatre), when undergoing an eligible procedure. In this context, unmasking of the cluster to their randomised group prior to participant recruitment is required for the purposes of training and delivery of the randomised allocation. In addition, there may be multiple units of exposure (e.g. operating rooms, teams, theatre lists) within a cluster representing a major source of variability, and many interdisciplinary team members are involved in the perioperative care pathway.

Despite these risks, strategies to minimise bias and imbalance have been poorly described and inconsistently reported to date [4, 11]. This paper describes strategies to minimise bias and imbalance by arm used in a global surgery cRCT and transparently reports their implementation and effectiveness.

## Methods

### Trial aims, design, and setting

ChEETAh was an international, multicentre, 2-arm, cRCT with an internal pilot [12]. It evaluated the use of separate sterile gloves and instruments before closing the abdominal wall to reduce the rate of surgical site infection in the 30 days after surgery. Patients undergoing any abdominal surgery, for any indication with an abdominal incision ≥ 5 cm were eligible, except for caesarean sections. The primary outcome was surgical site infection (SSI) at 30 days, based on a US Centers for Disease Control definition of SSI [13]. Overall, CHEETAH planned to recruit 12,800 patients in at least 64 clusters. UK ethical approval was obtained from the University of Birmingham International Research Ethics Committee. All individual participating countries obtained local or national ethical approval for ChEETAh in accordance with local requirements (available upon request). Individual patient-level consent for exposure to the intervention or control (routine practice) was deemed not to be required, so patients confirmed their consent (written or fingerprint) prior to discharge for inclusion in data collection at 30 days postoperatively.

Clusters were defined at the level of the hospital, from seven low- and middle-income countries (Benin, Ghana, India, Mexico, Nigeria, Rwanda, and South Africa). Hospitals were randomised (1:1) between (i) intervention (change of gloves and use of separate, sterile instruments) and (ii) current routine hospital practice (no change of gloves or use of separate, sterile instruments) *before* closing the abdominal wall. Low- and middle-income status was defined by the Development Assistance Committee (DAC) Official Development Assistance (ODA) list. Any cluster (hospital) in those LMICs that performed elective and/or emergency abdominal surgery and where glove and instrument change was not routine practice were eligible to participate. As the intervention and control require whole-team implementation, site investigators were not blinded, but patients were blinded to their randomisation status. This study was a pre-planned analysis of ChEETAh trial data to describe the strategies to minimise and monitor biases and chance imbalances and inform future cRCTs in surgery. DMC and TSC approval was obtained for the publication of this data.

### Trial structure and processes

In each randomised hospital and their pre-specified operating rooms, a bespoke local pathway was developed to recruit all eligible patients. Potentially eligible patients could be identified by any member of the surgical team (research nurse, clinical officer, surgeon in training, operating surgeon), either before, during or after surgery but before discharge.

### Pre-defined strategies to minimise bias and chance imbalances

**Fig. 1:**
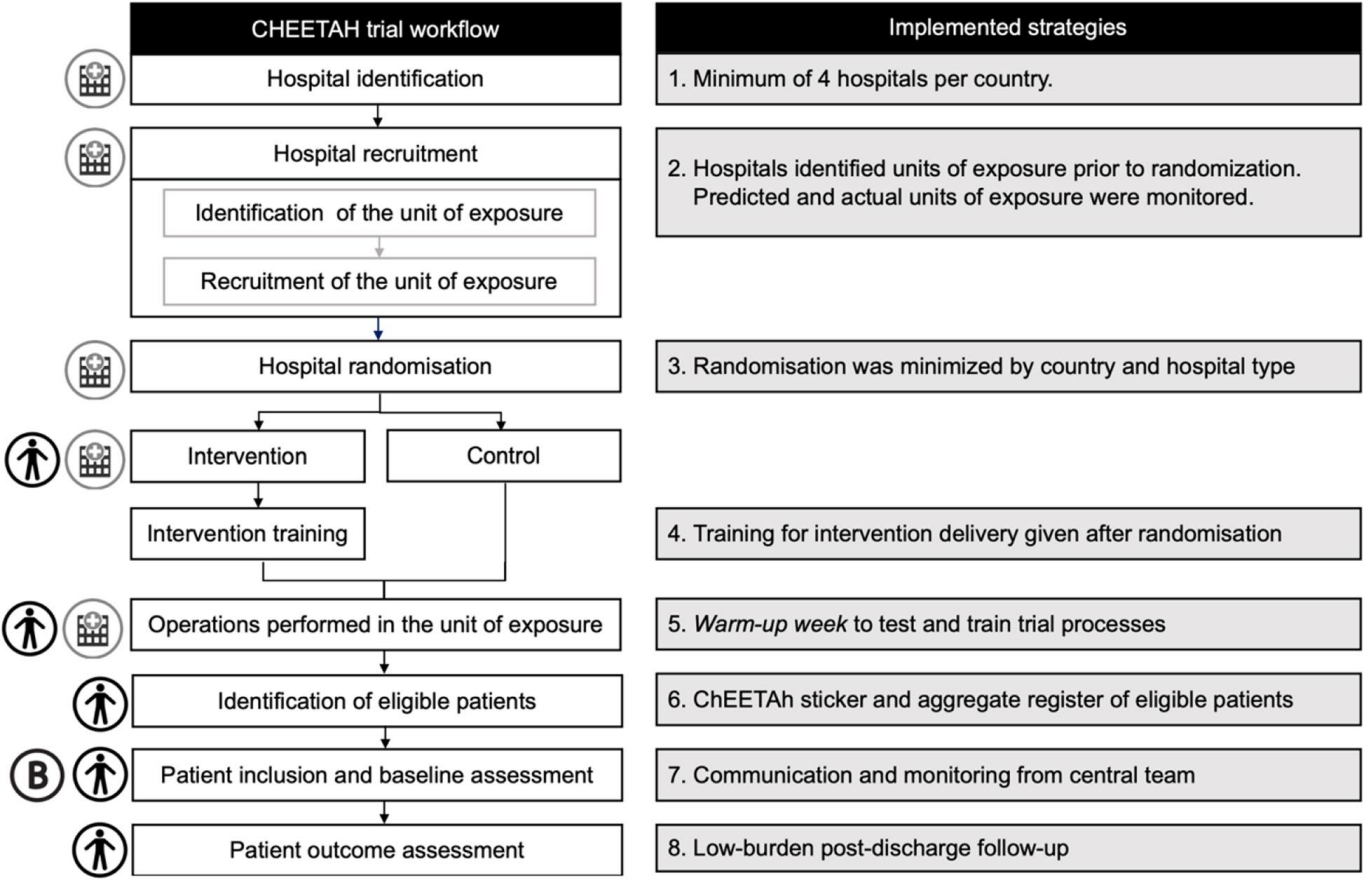
Flowchart of the trial processes and strategies to minimise bias and imbalance by arm

This cRCT protocol adopted eight strategies to monitor and minimise bias and imbalance. The strategies are ordered and aligned to the relevant stage of the trial pathway (Fig. 1). These focused on each of the three potential sources of bias based on case-mix variability: (1) hospitals; (2) units of exposure (operating theatres, surgical teams, and/or theatre lists); and (3) patients.

#### Strategy 1

Hospital-level: The protocol required a minimum of 4 hospitals randomised per participating LMIC to ensure balance in a number of clusters within each country.

#### Strategy 2

Unit of exposure level: Hospitals (clusters) were required to pre-specify their predicted participating units of exposure *prior to* randomisation (elective-only operating theatre/emergency-only operating theatre/elective and emergency (mixed) theatre). This was prospectively monitored, and any deviation in actual units of exposure post-randomisation were queried and recorded, with a direct intervention by the Trial Management Group (TMG) where feasible. This strategy aimed to prevent sites from modifying their planned case mix after knowledge of their randomised allocation. We report the proportion of hospitals that maintained their pre-specified operating theatre case mix overall and by randomised allocation.

#### Strategy 3

Hospital-level: Randomisation was minimised by country and by hospital type (i.e. hospital that accepts pre-operative referrals from other surgical teams (referral hospital) or not (non-referral hospital)) to force balance in randomisation allocation for these key characteristics. We reported the balance of intervention to control groups across the minimisation characteristics.

#### Strategy 4

Hospital-level: Hospitals and investigators were given a training on the intervention or control group allocation *after* randomisation to minimise contamination between the arms. Delivering training to hospitals prior to randomisation would have required the inclusion of information about the delivery of both the intervention and control groups. Training was conducted with multiple stakeholders across research hubs (national lead sites) and spokes (linked participating hospital), often across multiple theatre teams within each cluster. We reported the proportion of participating hospitals that received training after randomisation.

#### Strategy 5

Hospital-level: A dedicated ‘warm-up week’ was conducted by each hospital investigator team to establish and test processes for patient identification, including the use of the trial-specific register and the ChEETAh trial operation sticker (Additional file 1: Appendix C, D). We reported the number of clusters that completed a warm-up week.

#### Strategy 6

Patient-level: The identification of patients within clusters was ensured using a ChEETAh trial sticker in the clinical notes of all patients undergoing abdominal surgery in participating units of exposure and monitored with a dedicated in-theatre trial-specific register where patient eligibility and inclusion were both recorded. This information was held at sites and aggregated periodically, for both site monitoring against theatre logbooks and periodic reporting to the central co-ordinating team. The central team would then review the aggregate register (Additional file 1: Appendix E) and the cases uploaded onto REDCap, to identify any inappropriate patient exclusions and mitigate against resulting bias and imbalances. The number of eligible patients identified and included in the cRCT divided by the number of potentially eligible patients recorded in the aggregate register was used to calculate a case ascertainment rate summarised as a percentage with 95% confidence intervals.

#### Strategy 7

Hospital-level: Regular communication between the sites and the central coordinating team was maintained throughout the trial. This focused on monitoring key characteristics of patients: Patient level—age (≥ / < 18 years old), sex (male/female), urgency of operation (elective/emergency), operative approach (open midline/open non-midline), indication for surgery (malignant/benign).

These were reported monthly by the hospital to the TMG meeting to prospectively monitor and intervene in any hospitals where the quality assurance rules were not being followed and to the Data Monitoring Committee (DMC) and Trial Steering Committee (TSC) in regular reports. This strategy aimed to identify early any indication that units of exposure and patients may have been recruited selectively (i.e. unrepresentative sample, unusual or unexplained imbalance between the randomisation arms). We report the impact of our communication strategy in identifying and intervening on participating sites. This included routine central monitoring of the aggregate register, sharing lessons learned across the network and corrective and preventative measures around training and provision of additional guidance to all sites reinforcing several key trial processes (more details in Additional file 1: Appendix A).

#### Strategy 8

Patient-level: A pragmatic, low-burden post-discharge follow-up schedule was designed with support from patient and public representatives, to maximise feasibility and minimise selective outcome reporting in the trial. Refusal of consent for outcome assessment was reported overall across key risk subgroups and by trial arm.

### Data management and governance

No patient-level outcome data (e.g. SSI rates) was seen by the TMG during trial conduct, nor is it included in this publication. Clusters were pseudo-anonymised by the use of a hospital ID for presentation in this analysis. Reporting of this process was pre-defined in the published study protocol [12]. Summary data were described using summary statistics in Stata V17.0 (Stata Corporation).

## Results

Data were included in this analysis from 10,686 patients undergoing surgery in 70 hospitals in four countries. Summary data is presented below for each of the eight strategies to monitor and minimise bias and imbalances (Fig. 1).

### Strategy 1

Six countries had met their minimum requirement for a number of participating centres (India, 21 hospitals; Nigeria, 16 hospitals; Rwanda, 12 hospitals; Ghana, 10 hospitals; Benin, 5 hospitals; Mexico, 4 hospitals) and one was below this target (South Africa, 2 hospitals).

### Strategy 2

**Fig. 2:**
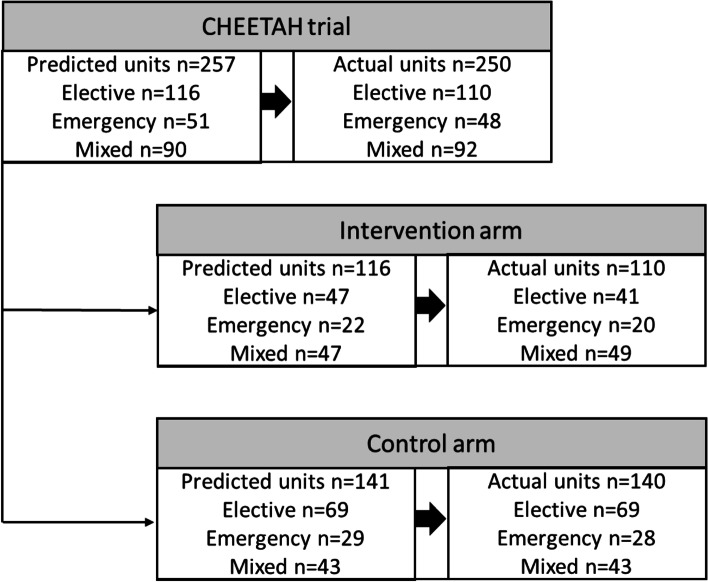
Flowchart describing the predicted and actual theatres participating in the ChEETAh cRCT

Of all hospitals included in the analysis, 61 of 70 (87.1%, 95% CI 77.0 to 93.9%) collected data from patients in the same number of elective-only, emergency-only and mixed elective-emergency units of exposure that they had predicted prior to randomisation (Fig. S4). There were no occasions where these hospitals changed their specified elective or emergency units of exposure (i.e. swapping one elective theatre for another in the same hospital when examined by randomisation allocation (Fig. 2)); there was appropriate balance between the intervention and control arm in the number of elective (37.3%, 95% CI 28.2 to 47.0% [41/110] versus 49.3%, 95% CI 40.7 to 57.9% [69/140]), emergency (18.2%, 95% CI 11.5 to 26.7% [20/110] versus 20.0%, 95% CI 13.7 to 27.6% [28/140]) and mixed elective and emergency theatres (44.5%, 95% CI 35.1 to 54.3% [49/110] versus 30.7%, 95% CI 23.2 to 39.1% [43/140]).

### Strategy 3

Randomisation was generally well-balanced at a hospital level in Benin (4 interventions versus 1 control), Ghana (4 interventions versus 6 controls), India (11 interventions versus 10 controls), Mexico (2 interventions versus 2 controls), Nigeria (8 interventions versus 8 controls), Rwanda (7 interventions versus 5 controls) and South Africa (1 intervention versus 1 control) in line with the minimisation criteria. Equally, randomisation was well-balanced between referral (30 interventions versus 27 controls) and non-referral hospitals (7 interventions versus 6 controls).

### Strategy 4

All sites completed investigator training during virtual site initiation, with a median of 10 (IQR: 5–19) investigators present per virtual training session. All sites completed the online training modules with a mean of 9 investigators per site.

### Strategy 5

The warm-up week was successfully conducted by all sites (70/70), and feedback was used to refine trial processes.

### Strategy 6

Training on the use and relevance of the ChEETAh sticker was delivered to all participating hospitals (70/70). The aggregate register was maintained at all sites (70/70), with an overall inclusion of 98.1% (10,686/10,894, 95% CI 97.8 to 98.3%) of the eligible patients which was balanced by randomised allocation (98.6%, 95% CI 98.3 to 98.9% control vs 97.5%, 95% CI 97.1 to 97.9% intervention). A summary is presented in Additional file 1: Table S1. A median of 0 eligible patients (IQR: 0–1) was missed from inclusion. Routine monitoring of the aggregate register against the patient-level data uploaded on REDCap was conducted, allowing prompt identification of missed patients and further site training on recruitment processes.

### Strategy 7

A summary of patient-level monitoring measures by cluster is shown in Additional file 1: Table 2, demonstrating the between-cluster variability in patient characteristics. A summary of monitoring measure by trial arm is presented in Table 1. Chance imbalances arose between the intervention and control groups in the proportions of patients who were < 18 years (8.9% versus 5.9%), ASA grade IV or V (7.2% versus 4.2%), emergency surgery (56.8% versus 46.6%, Fig. S1), benign disease (80.4% versus 59.4%), no WHO Surgical Safety Checklist (3.4% versus 0.2%) and open non-midline surgery (30.5% versus 19.1%). Chance imbalances reduced over time; an example is provided for the urgency of surgery in Fig. S2. Figure S3 demonstrates the interplay between the urgency of surgery, the type of incision and surgical indication overall. Non-midline incisions were more frequent in elective surgery (27.2% [416/1635] vs 21.7% [352/1527] in emergency surgery) and were more often performed for benign surgical indications. These key patient characteristics were monitored centrally and reported at the TMG meetings, with swift action from the trials unit whenever biases were suspected. As an example, one site was enquired about inconsistencies in the aggregate register, uploaded data and theatre logbooks, admitting that some patients were missed. Lessons learned were disseminated across all hubs and spokes to avoid similar situations (see Additional file 1: Appendix F for more details on communication with the central management team).

**Table 1:** Baseline key characteristics of the patients included in the intervention and control arms

Factor	Routine practice, *n* = 5640	Change of gloves and instruments, *n* = 5046
**Age**		
< 18 years	484 (8.6)	522 (10.3)
≥ 18 years	5155 (91.4)	4524 (89.7)
**ASA grade**		
Grade I	2539 (45.0)	2600 (51.5)
Grade II	2086 (37.0)	1571 (31.1)
Grade III	757 (13.4)	735 (14.6)
Grade IV	158 (2.8)	121 (2.4)
Grade V	99 (1.8)	19 (0.4)
**Timing of surgery**		
Elective	2486 (44.1)	2603 (51.6)
Emergency	3153 (55.9)	2443 (48.4)
**Indication**		
Malignant disease	860 (15.2)	955 (18.9)
Benign disease	4506 (79.9)	3812 (75.5)
Trauma	274 (4.9)	279 (5.5)
**WHO surgical safety checklist**		
Yes	5387 (95.5)	4882 (96.8)
No	252 (4.5)	163 (3.2)
**Operative approach**		
Open—midline	3344 (59.3)	3325 (65.9)
Open—non-midline	2172 (38.5)	1605 (31.8)
Laparoscopic	38 (0.7)	56 (1.1)
Laparoscopic converted to open	86 (1.5)	60 (1.2)

### Strategy 8

Overall, 0.4% patients (41/9187, 95% CI 0.3 to 0.6) refused consent for outcome assessment. This was balanced by trial arm (0.5% control vs 0.4% intervention) and across key risk subgroups (Additional file 1: Table S3).

## Discussion

This study evaluated eight strategies to monitor and minimise bias and imbalances within an international cluster randomised controlled trial in surgery. Hospital-level strategies contributed to balance in unit of exposure level strategies, which in turn contributed to balance in patient-level strategies. Although all the strategies were delivered with success, chance imbalances in monitoring measures such as elective versus emergency operations, midline versus non-midline incisions and surgery for benign versus malignant indications were observed. Monitoring data were regularly reviewed by the TMG, with intervention at a site level where required. The diverse delivery network demonstrates the generalisability of future analyses of the primary outcome measure. Our data reflect that cRCTs are prone to chance imbalances at multiple stages and a transparent reporting of mitigation strategies is crucial for an adequate interpretation of the trial results. Pre-planning for chance imbalance in cRCT statistical analysis plans should be considered paramount.

cRCTs have increasingly been recognised in surgery and interventional specialties as a methodology for evaluating complex and/or behavioural change interventions in operating theatres [1–3, 14]. Operating theatres are multi-professional, multi-specialty environments where culture change is often required to empower practice change and uptake of evidence-based practice is slow [15]. Cluster methods, learning from concepts in implementation and behavioural science, have significant potential in both evaluating late-phase interventions and encouraging sustainable adoption where benefit is observed. This may be particularly relevant to resource-constrained settings, where contextually sensitive interventions require deep co-development, and the ability to scale across networks is key [16]. However, cluster trials in surgery are challenging. First, clusters are often defined at a hospital level but significant within-cluster heterogeneity exists both in the unit of exposure (operating theatre, surgical team or theatre list) and patient-level case mix (urgency and types of operation). Although patients undergoing elective and emergency surgery are expected to have distinct characteristics and outcomes [17–19], the arm imbalances observed in the ChEETAh trial are expected as with any cluster trials. With appropriate adjustments for key confounders in statistical analysis, this will limit the compromise of the internal validity of the results. Second, patients cannot be recruited upfront before randomisation as in the case chronic disease management, so recruitment is performed after randomisation [20]. In the ChEETAh trial, 97% of the hospitals maintained their case mix of emergency and elective theatres, demonstrating very little recruitment bias after randomisation. The main model of this trial will adjust for minimisation factors (country and type of hospital) as well as key confounders which include both hospital-level and patient-level covariates.

Previous authors have described similar methods for cRCTs but have described application to outpatient settings or chronic disease and have limited applicability to the surgical settings (i.e. one-off intervention exposure, multi-level within and between cluster variability) [20]. Chance imbalances are expected in cRCTs and adjustment for the key cluster- and patient-level variables whilst accounting for clustering is essential during data analysis [8]. These statistical techniques however cannot account for unmeasured confounding so should be seen as part of a whole-trial approach to bias mitigation, rather than standing alone [11]. We observed here that chance imbalances between key risk variables decreased over time (i.e. as sample size increased); this has been previously observed in meta-analyses of cRCTs [21] which suggests that patient-level imbalance is expected in cRCTs.

As this trial did not require patient-level consent for exposure to the intervention, but did for outcome assessment, the risk of bias from the refusal of consent was low. We worked with patient and community partners to design a pragmatic follow-up schedule [22] that was low burden (completed at a single time point) and collected outcome data from electronic health records where feasible (e.g. reoperation) [23]. Equally, we had no centres that recruited no patients so would be excluded from an intention-to-treat analysis [20], nor was there a risk of cluster ‘migration’ (where participants move out or into one cluster to/from another) as with some trials in chronic disease. Patients were the only blinded party in this trial, as the operating teams were delivering the intervention and outcome assessors were likely to be aware of the hospital allocation. Cluster randomised trials in surgery are unique, and designing them requires multifaceted considerations of different issues that may warrant such strategies to minimise risk of bias as compared to other fields. For instance, there are weekend and night operations, and decisions on patient inclusion into trials are difficult. Further blinding in surgery is not entirely feasible. This trial has been co-developed with stakeholders from LMICs, through face-to-face meetings and a formal Delphi process, to ensure its relevance. Both the interventions and design of research were judged to be applicable to settings in lower-resource countries, as judged by frontline surgeons. Finally, we had many clusters (*N* = 70) and a randomisation-minimisation algorithm, so allocation concealment was easily maintained (i.e. unlikely that new clusters would be able to anticipate their randomised allocation). Future trials where there would be a risk of these alternative sources of bias should consider including methods to monitor and minimise these during planning and implementation [24].

This study had limitations. First, we were unable to fully account for the risk of recruitment bias using the methods described [25]. However, by using consecutive sampling of eligible patients and monitoring for refusal of consent are actively aware of this, and with interpretation of the full trial results with this caveat. Second, imbalances persisted in certain patient-level variables between randomisation arms. These decreased as sample size increased (suggesting chance imbalance only). Future meta-analyses of cRCTs in this area are warranted to fully assess the effectiveness of these strategies in mitigating against selection bias in cRCTs. Third, we specifically highlight chance imbalance and attrition bias as key causes of differential misclassification in cRCTs but other sources of bias exist (performance bias, measurement bias, dilution bias) that we have not considered here. Fourth, whilst we have focussed on surgical cRCTs, the findings may be applicable to other interventional and procedural specialties where differences in outcomes can occur related to the procedural performer, environment and technique in addition to patient-related factors. We encourage other investigators to explore these concepts across other specialty areas.

Future guidance on the delivery and reporting of cRCTs in surgery and other areas of knowledge should incorporate strategies similar to these to mitigate against bias and imbalance by arm. The ChEETAh trial provides a structured approach which was implemented successfully across diverse settings and can be flexibly applied to future cRCTs.

## Supplementary Information

**Additional file 1:** **Fig. S1.** Balance of elective and emergency surgery by trial arm. **Fig. S2.** Changes in imbalance by arm of urgency of surgery over time. **Fig. S3.** Flowchart of key surgery characteristics overall. **Fig. S4.** Number of actual and predicted units of exposure per hospital. **Table S1.** Number of eligible patients included (patient-level analysis). **Table S2.** Baseline characteristics of included patients (N=, %) (hospital-level analysis). **Table S3.** Refusal of consent for outcome assessment (patient-level analysis). **Table S5.** Mapping strategy domains to Cochrane risk of bias tool. **Appendix A.** Author list. **Appendix B.** Strategies following identification of potential sources of bias. **Appendix C.** ChEETAh trial-specific register. Appendix D. ChEETAh trial operation sticker. **Appendix E.** ChEETAh aggregate register. **Appendix F.** ChEETAh Patient Pathway Flowchart.
