# Supplementary material for: Strategies to minimise and monitor biases and imbalances by arm in surgical cluster randomised trials: evidence from ChEETAh, a trial in seven low- and middle-income countries
Source: Trials. 2023 Apr 5;24:259. doi: 10.1186/s13063-022-06852-2 (PMC10077601; doi:10.1186/s13063-022-06852-2)

**Supplementary figure 1.** Balance of elective and emergency surgery by trial arm.


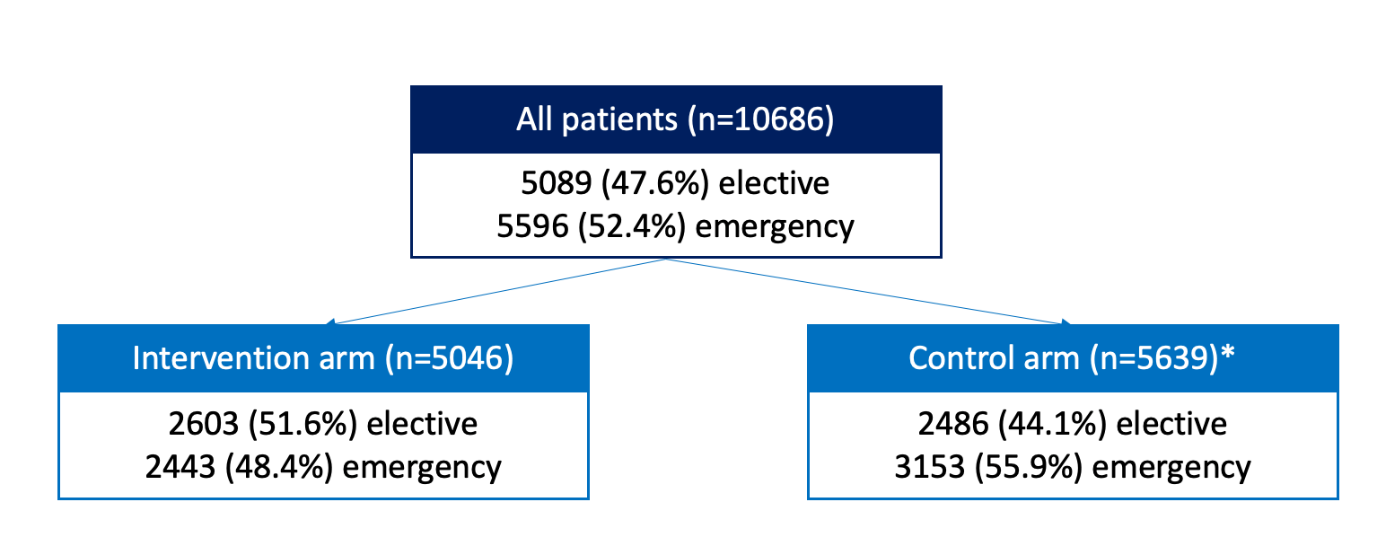


* 1 missing urgency of operation

**Supplementary figure 2.** Changes in imbalance by arm of urgency of surgery over time

**Supplement figure 3.** Flowchart of key surgery characteristics overall*
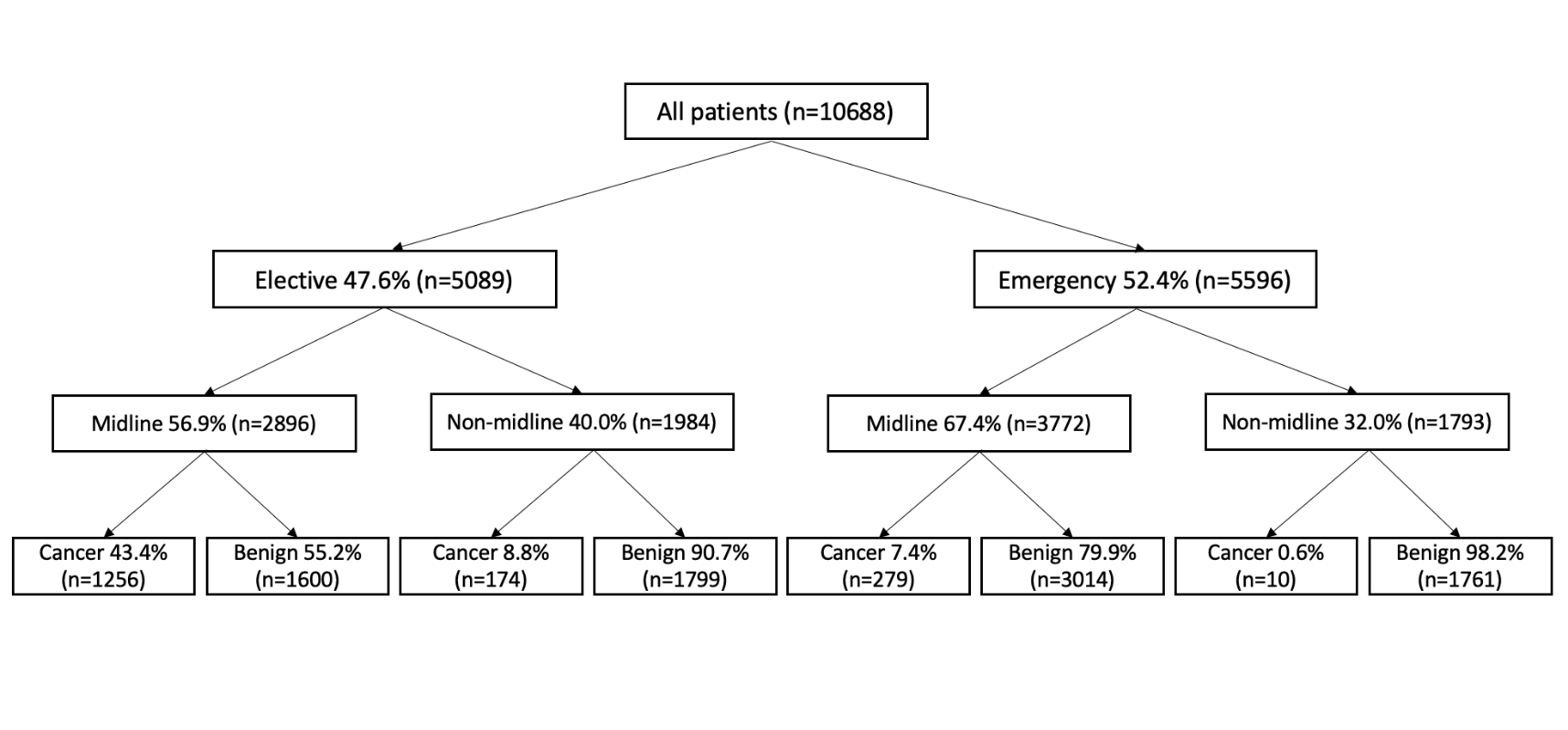
*

**Supplementary Figure 4.** Number of actual and predicted units of exposure per hospital.*
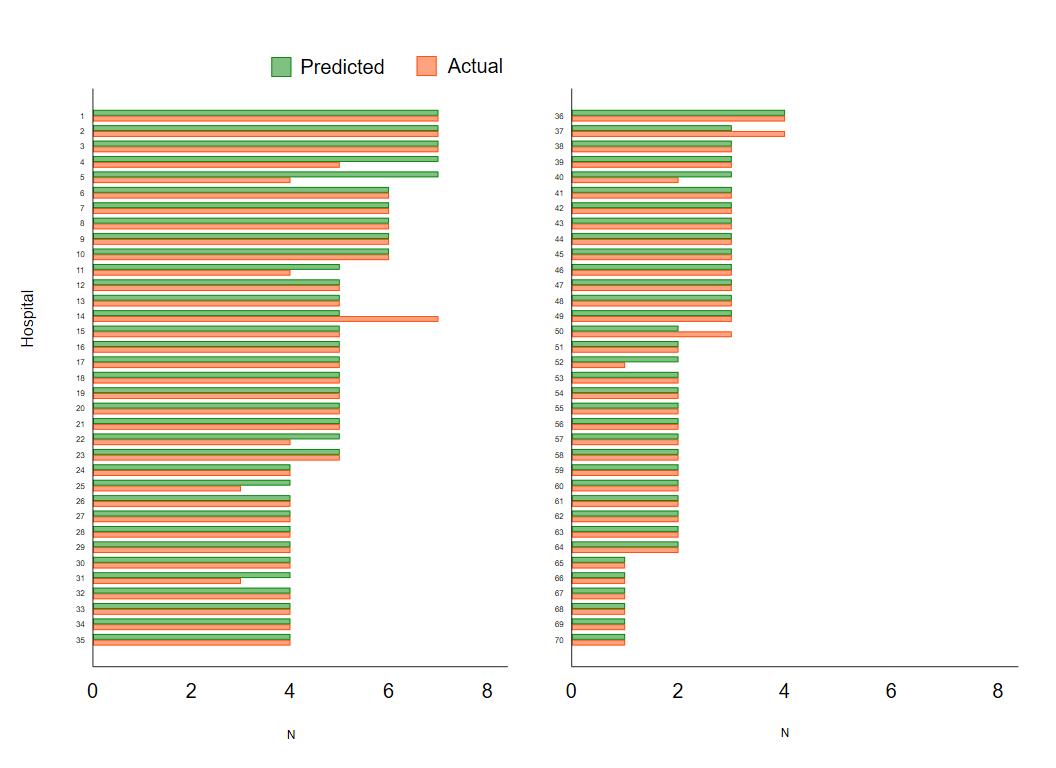
*

Charts display the number of predicted (green) and actually included (orange) units of exposure per hospital.

**Supplementary table 1.** Number of eligible patients included *(patient-level analysis)*

| **Hospital ID** | **Country** | **Number of eligible patients** | **Number of patients included** | **% of patients included** | **Number of patients missed from inclusion** |
| --- | --- | --- | --- | --- | --- |
| **1** | Benin | 53 | 53 | 100 | 0 |
| **2** | Benin | 18 | 18 | 100 | 0 |
| **3** | Benin | 5 | 5 | 100 | 0 |
| **4** | Benin | 90 | 89 | 98.89 | 1 |
| **5** | Benin | 40 | 40 | 100 | 0 |
| **6** | Ghana | 201 | 201 | 100 | 0 |
| **7** | Ghana | 71 | 66 | 92.96 | 5 |
| **8** | Ghana | 177 | 177 | 100 | 0 |
| **9** | Ghana | 91 | 91 | 100 | 0 |
| **10** | Ghana | 223 | 220 | 98.65 | 3 |
| **11** | Ghana | 234 | 216 | 92.31 | 18 |
| **12** | Ghana | 202 | 202 | 100 | 0 |
| **13** | Ghana | 20 | 19 | 95 | 1 |
| **14** | Ghana | 187 | 187 | 100 | 0 |
| **15** | Ghana | 185 | 185 | 100 | 0 |
| **16** | India | 300 | 300 | 100 | 0 |
| **17** | India | 226 | 220 | 97.35 | 6 |
| **18** | India | 140 | 135 | 96.43 | 5 |
| **19** | India | 220 | 220 | 100 | 0 |
| **20** | India | 133 | 133 | 100 | 0 |
| **21** | India | 80 | 71 | 88.75 | 9 |
| **22** | India | 204 | 200 | 98.04 | 4 |
| **23** | India | 220 | 220 | 100 | 0 |
| **24** | India | 238 | 238 | 100 | 0 |
| **25** | India | 71 | 71 | 100 | 0 |
| **26** | India | 223 | 215 | 96.41 | 8 |
| **27** | India | 67 | 66 | 98.51 | 1 |
| **28** | India | 206 | 205 | 99.51 | 1 |
| **29** | India | 258 | 252 | 97.67 | 6 |
| **30** | India | 220 | 220 | 100 | 0 |
| **31** | India | 220 | 220 | 100 | 0 |
| **32** | India | 62 | 62 | 100 | 0 |
| **33** | India | 67 | 67 | 100 | 0 |
| **34** | India | 246 | 246 | 100 | 0 |
| **35** | India | 96 | 95 | 98.96 | 1 |
| **36** | India | 217 | 202 | 93.09 | 15 |
| **37** | Mexico | 8 | 8 | 100 | 0 |
| **38** | Mexico | 144 | 144 | 100 | 0 |
| **39** | Mexico | 3 | 3 | 100 | 0 |
| **40** | Mexico | 34 | 34 | 100 | 0 |
| **41** | Nigeria | 154 | 154 | 100 | 0 |
| **42** | Nigeria | 118 | 102 | 86.44 | 16 |
| **43** | Nigeria | 20 | 20 | 100 | 0 |
| **44** | Nigeria | 33 | 33 | 100 | 0 |
| **45** | Nigeria | 40 | 40 | 100 | 0 |
| **46** | Nigeria | 220 | 220 | 100 | 0 |
| **47** | Nigeria | 220 | 220 | 100 | 0 |
| **48** | Nigeria | 220 | 220 | 100 | 0 |
| **49** | Nigeria | 118 | 118 | 100 | 0 |
| **50** | Nigeria | 20 | 20 | 100 | 0 |
| **51** | Nigeria | 202 | 202 | 100 | 0 |
| **52** | Nigeria | 97 | 97 | 100 | 0 |
| **53** | Nigeria | 147 | 147 | 100 | 0 |
| **54** | Nigeria | 224 | 220 | 98.21 | 4 |
| **55** | Nigeria | 77 | 77 | 100 | 0 |
| **56** | Nigeria | 35 | 35 | 100 | 0 |
| **57** | Rwanda | 284 | 284 | 100 | 0 |
| **58** | Rwanda | 9 | 9 | 100 | 0 |
| **59** | Rwanda | 300 | 300 | 100 | 0 |
| **60** | Rwanda | 300 | 300 | 100 | 0 |
| **61** | Rwanda | 220 | 220 | 100 | 0 |
| **62** | Rwanda | 319 | 300 | 94.04 | 19 |
| **63** | Rwanda | 300 | 300 | 100 | 0 |
| **64** | Rwanda | 300 | 300 | 100 | 0 |
| **65** | Rwanda | 221 | 220 | 99.55 | 1 |
| **66** | Rwanda | 182 | 166 | 91.21 | 16 |
| **67** | Rwanda | 300 | 300 | 100 | 0 |
| **68** | Rwanda | 29 | 26 | 89.66 | 3 |
| **69** | South Africa | 252 | 200 | 79.37 | 52 |
| **70** | South Africa | 236 | 220 | 93.22 | 16 |

**Supplementary table 2.** Baseline characteristics of included patients (N=, %) *(hospital-level analysis)*

| **Hospital ID** | **Country** | **Age ≥18 years** | **Gender, Female** | **Elective** | **Clean-**  **contaminated** | **Contaminated** | **Dirty** | **Malignant disease** | **Open-Midline** | **Open Non-midline** |
| --- | --- | --- | --- | --- | --- | --- | --- | --- | --- | --- |
| **1** | Benin | 44 (83.0) | 23 (43.4) | 2 (3.8) | 21 (39.6) | 1 (1.9) | 31 (58.5) | 1 (1.9) | 14 (26.4) | 39 (73.6) |
| **2** | Benin | 10 (55.6) | 9 (50.0) | 4 (22.2) | 2 (11.1) | 5 (27.8) | 11 (61.1) | 0 (0.0) | 17 (94.4) | 1 (5.6) |
| **3** | Benin | 3 (60.0) | 1 (20.0) | 2 (40.0) | 2 (40.0) | 1 (20.0) | 2 (40.0) | 0 (0.0) | 4 (80.0) | 1 (20.0) |
| **4** | Benin | 63 (70.8) | 35 (39.3) | 5 (5.6) | 40 (44.9) | 0 (0.0) | 49 (55.1) | 1 (1.1) | 24 (27.0) | 65 (73.0) |
| **5** | Benin | 32 (80.0) | 24 (60.0) | 0 (0.0) | 14 (35.0) | 21 (52.5) | 5 (12.5) | 0 (0.0) | 10 (25.0) | 30 (75.0) |
| **6** | Ghana | 174 (86.6) | 70 (34.8) | 7 (3.5) | 57 (28.4) | 74 (36.8) | 70 (34.8) | 12 (6.0) | 102 (50.7) | 99 (49.3) |
| **7** | Ghana | 61 (92.4) | 49 (74.2) | 6 (9.1) | 27 (40.9) | 29 (43.9) | 10 (15.2) | 2 (3.0) | 29 (43.9) | 37 (56.1) |
| **8** | Ghana | 161 (91.0) | 79 (44.6) | 73 (41.2) | 109 (61.6) | 29 (16.4) | 39 (22.0) | 21 (11.9) | 134 (75.7) | 41 (23.2) |
| **9** | Ghana | 76 (83.5) | 27 (29.7) | 21 (23.1) | 40 (44.0) | 8 (8.8) | 43 (47.3) | 0 (0.0) | 59 (64.8) | 32 (35.2) |
| **10** | Ghana | 183 (83.2) | 108 (49.1) | 7 (3.2) | 88 (40.0) | 89 (40.5) | 43 (19.5) | 0 (0.0) | 150 (68.2) | 70 (31.8) |
| **11** | Ghana | 178 (82.4) | 77 (35.6) | 26 (12.0) | 121 (56.0) | 71 (32.9) | 24 (11.1) | 27 (12.5) | 187 (86.6) | 29 (13.4) |
| **12** | Ghana | 171 (84.7) | 70 (34.7) | 55 (27.2) | 60 (29.7) | 64 (31.7) | 78 (38.6) | 55 (27.2) | 140 (69.3) | 61 (30.2) |
| **13** | Ghana | 10 (52.6) | 4 (21.1) | 3 (15.8) | 4 (21.1) | 2 (10.5) | 13 (68.4) | 0 (0.0) | 15 (78.9) | 4 (21.1) |
| **14** | Ghana | 171 (91.4) | 61 (32.6) | 1 (0.5) | 102 (54.5) | 71 (38.0) | 14 (7.5) | 4 (2.1) | 131 (70.1) | 56 (29.9) |
| **15** | Ghana | 150 (81.1) | 63 (34.1) | 16 (8.6) | 34 (18.4) | 50 (27.0) | 101 (54.6) | 9 (4.9) | 134 (72.4) | 51 (27.6) |
| **16** | India | 298 (99.3) | 136 (45.3) | 210 (70.0) | 298 (99.3) | 1 (0.3) | 1 (0.3) | 218 (72.7) | 298 (99.3) | 2 (0.7) |
| **17** | India | 204 (92.7) | 131 (59.5) | 146 (66.4) | 163 (74.1) | 21 (9.5) | 36 (16.4) | 106 (48.2) | 143 (65.0) | 57 (25.9) |
| **18** | India | 131 (97.0) | 81 (60.0) | 80 (59.3) | 74 (54.8) | 42 (31.1) | 19 (14.1) | 2 (1.5) | 52 (38.5) | 70 (51.9) |
| **19** | India | 205 (93.2) | 66 (30.0) | 62 (28.2) | 55 (25.0) | 53 (24.1) | 112 (50.9) | 19 (8.6) | 172 (78.2) | 47 (21.4) |
| **20** | India | 128 (96.2) | 82 (61.7) | 102 (76.7) | 96 (72.2) | 26 (19.5) | 11 (8.3) | 6 (4.5) | 47 (35.3) | 86 (64.7) |
| **21** | India | 67 (94.4) | 47 (66.2) | 48 (67.6) | 63 (88.7) | 2 (2.8) | 6 (8.5) | 2 (2.8) | 22 (31.0) | 21 (29.6) |
| **22** | India | 188 (94.0) | 114 (57.0) | 88 (44.0) | 111 (55.5) | 65 (32.5) | 24 (12.0) | 18 (9.0) | 124 (62.0) | 74 (37.0) |
| **23** | India | 220 (100.0) | 134 (60.9) | 171 (77.7) | 185 (84.1) | 26 (11.8) | 9 (4.1) | 130 (59.1) | 195 (88.6) | 15 (6.8) |
| **24** | India | 228 (95.8) | 128 (53.8) | 221 (92.9) | 218 (91.6) | 17 (7.1) | 3 (1.3) | 77 (32.4) | 185 (77.7) | 33 (13.9) |
| **25** | India | 60 (84.5) | 31 (43.7) | 16 (22.5) | 21 (29.6) | 31 (43.7) | 19 (26.8) | 3 (4.2) | 30 (42.3) | 40 (56.3) |
| **26** | India | 196 (91.2) | 107 (49.8) | 108 (50.2) | 100 (46.5) | 41 (19.1) | 74 (34.4) | 2 (0.9) | 85 (39.5) | 129 (60.0) |
| **27** | India | 66 (100.0) | 45 (68.2) | 66 (100.0) | 66 (100.0) | 0 (0.0) | 0 (0.0) | 66 (100.0) | 62 (93.9) | 4 (6.1) |
| **28** | India | 198 (96.6) | 125 (61.0) | 181 (88.3) | 183 (89.3) | 22 (10.7) | 0 (0.0) | 6 (2.9) | 80 (39.0) | 123 (60.0) |
| **29** | India | 241 (95.6) | 147 (58.3) | 163 (64.7) | 203 (80.6) | 39 (15.5) | 10 (4.0) | 10 (4.0) | 124 (49.2) | 128 (50.8) |
| **30** | India | 199 (90.5) | 127 (57.7) | 95 (43.2) | 152 (69.1) | 19 (8.6) | 49 (22.3) | 10 (4.5) | 96 (43.6) | 108 (49.1) |
| **31** | India | 202 (91.8) | 71 (32.3) | 93 (42.3) | 48 (21.8) | 66 (30.0) | 106 (48.2) | 10 (4.5) | 144 (65.5) | 68 (30.9) |
| **32** | India | 0 (0.0) | 31 (50.0) | 33 (53.2) | 35 (56.5) | 20 (32.3) | 7 (11.3) | 9 (14.5) | 18 (29.0) | 40 (64.5) |
| **33** | India | 66 (98.5) | 46 (68.7) | 57 (85.1) | 59 (88.1) | 6 (9.0) | 2 (3.0) | 26 (38.8) | 47 (70.1) | 17 (25.4) |
| **34** | India | 246 (100.0) | 102 (41.5) | 168 (68.3) | 19 (7.7) | 222 (90.2) | 5 (2.0) | 135 (54.9) | 173 (70.3) | 53 (21.5) |
| **35** | India | 89 (93.7) | 36 (37.9) | 51 (53.7) | 43 (45.3) | 24 (25.3) | 28 (29.5) | 14 (14.7) | 53 (55.8) | 31 (32.6) |
| **36** | India | 202 (100.0) | 79 (39.1) | 184 (91.1) | 195 (96.5) | 7 (3.5) | 0 (0.0) | 198 (98.0) | 152 (75.2) | 14 (6.9) |
| **37** | Mexico | 0 (0.0) | 6 (75.0) | 1 (12.5) | 6 (75.0) | 2 (25.0) | 0 (0.0) | 1 (12.5) | 1 (12.5) | 7 (87.5) |
| **38** | Mexico | 143 (100.0) | 73 (51.0) | 126 (87.5) | 132 (91.7) | 12 (8.3) | 0 (0.0) | 58 (40.3) | 63 (43.8) | 69 (47.9) |
| **39** | Mexico | 3 (100.0) | 2 (66.7) | 0 (0.0) | 2 (66.7) | 1 (33.3) | 0 (0.0) | 0 (0.0) | 3 (100.0) | 0 (0.0) |
| **40** | Mexico | 0 (0.0) | 10 (29.4) | 9 (26.5) | 34 (100.0) | 0 (0.0) | 0 (0.0) | 0 (0.0) | 2 (5.9) | 32 (94.1) |
| **41** | Nigeria | 120 (77.9) | 88 (57.1) | 76 (49.4) | 83 (53.9) | 39 (25.3) | 32 (20.8) | 20 (13.0) | 70 (45.5) | 84 (54.5) |
| **42** | Nigeria | 75 (73.5) | 51 (50.0) | 47 (46.1) | 71 (69.6) | 19 (18.6) | 12 (11.8) | 12 (11.8) | 53 (52.0) | 48 (47.1) |
| **43** | Nigeria | 14 (70.0) | 13 (65.0) | 15 (75.0) | 15 (75.0) | 0 (0.0) | 5 (25.0) | 0 (0.0) | 10 (50.0) | 10 (50.0) |
| **44** | Nigeria | 28 (84.8) | 23 (69.7) | 16 (48.5) | 16 (48.5) | 8 (24.2) | 9 (27.3) | 1 (3.0) | 19 (57.6) | 14 (42.4) |
| **45** | Nigeria | 37 (92.5) | 19 (47.5) | 30 (75.0) | 32 (80.0) | 5 (12.5) | 3 (7.5) | 6 (15.0) | 19 (47.5) | 21 (52.5) |
| **46** | Nigeria | 199 (90.5) | 146 (66.4) | 123 (55.9) | 147 (66.8) | 40 (18.2) | 33 (15.0) | 43 (19.5) | 160 (72.7) | 54 (24.5) |
| **47** | Nigeria | 169 (76.8) | 125 (56.8) | 85 (38.6) | 97 (44.1) | 21 (9.5) | 102 (46.4) | 19 (8.6) | 78 (35.5) | 142 (64.5) |
| **48** | Nigeria | 196 (89.1) | 162 (73.6) | 164 (74.5) | 176 (80.0) | 16 (7.3) | 28 (12.7) | 48 (21.8) | 146 (66.4) | 72 (32.7) |
| **49** | Nigeria | 98 (83.1) | 69 (58.5) | 47 (39.8) | 52 (44.1) | 26 (22.0) | 40 (33.9) | 27 (22.9) | 72 (61.0) | 46 (39.0) |
| **50** | Nigeria | 17 (85.0) | 10 (50.0) | 18 (90.0) | 19 (95.0) | 1 (5.0) | 0 (0.0) | 1 (5.0) | 6 (30.0) | 14 (70.0) |
| **51** | Nigeria | 182 (90.1) | 138 (68.3) | 109 (54.0) | 135 (66.8) | 36 (17.8) | 31 (15.3) | 24 (11.9) | 106 (52.5) | 96 (47.5) |
| **52** | Nigeria | 91 (93.8) | 60 (61.9) | 53 (54.6) | 69 (71.1) | 12 (12.4) | 16 (16.5) | 19 (19.6) | 44 (45.4) | 53 (54.6) |
| **53** | Nigeria | 131 (89.1) | 90 (61.6) | 90 (61.6) | 101 (68.7) | 30 (20.4) | 16 (10.9) | 34 (23.1) | 85 (57.8) | 61 (41.5) |
| **54** | Nigeria | 190 (86.4) | 145 (65.9) | 102 (46.4) | 96 (43.6) | 87 (39.5) | 37 (16.8) | 16 (7.3) | 206 (93.6) | 13 (5.9) |
| **55** | Nigeria | 65 (84.4) | 45 (58.4) | 32 (41.6) | 56 (72.7) | 13 (16.9) | 8 (10.4) | 8 (10.4) | 36 (46.8) | 38 (49.4) |
| **56** | Nigeria | 30 (85.7) | 18 (51.4) | 15 (42.9) | 19 (54.3) | 13 (37.1) | 3 (8.6) | 3 (8.6) | 21 (60.0) | 14 (40.0) |
| **57** | Rwanda | 276 (97.2) | 265 (93.3) | 217 (76.4) | 275 (96.8) | 2 (0.7) | 7 (2.5) | 4 (1.4) | 213 (75.0) | 71 (25.0) |
| **58** | Rwanda | 9 (100.0) | 5 (55.6) | 4 (44.4) | 6 (66.7) | 2 (22.2) | 1 (11.1) | 2 (22.2) | 8 (88.9) | 1 (11.1) |
| **59** | Rwanda | 260 (86.7) | 117 (39.0) | 131 (43.7) | 182 (60.7) | 34 (11.3) | 84 (28.0) | 49 (16.3) | 272 (90.7) | 25 (8.3) |
| **60** | Rwanda | 274 (91.3) | 117 (39.0) | 137 (45.7) | 216 (72.0) | 52 (17.3) | 32 (10.7) | 83 (27.7) | 258 (86.0) | 38 (12.7) |
| **61** | Rwanda | 216 (98.2) | 197 (89.5) | 118 (53.6) | 184 (83.6) | 17 (7.7) | 19 (8.6) | 3 (1.4) | 111 (50.5) | 109 (49.5) |
| **62** | Rwanda | 293 (97.7) | 253 (84.3) | 137 (45.7) | 233 (77.7) | 25 (8.3) | 42 (14.0) | 16 (5.3) | 124 (41.3) | 176 (58.7) |
| **63** | Rwanda | 274 (91.3) | 203 (67.7) | 105 (35.0) | 191 (63.7) | 70 (23.3) | 39 (13.0) | 6 (2.0) | 160 (53.3) | 140 (46.7) |
| **64** | Rwanda | 282 (94.0) | 230 (76.7) | 82 (27.3) | 196 (65.3) | 52 (17.3) | 52 (17.3) | 21 (7.0) | 140 (46.7) | 160 (53.3) |
| **65** | Rwanda | 209 (95.0) | 183 (83.2) | 102 (46.4) | 187 (85.0) | 26 (11.8) | 7 (3.2) | 6 (2.7) | 111 (50.5) | 109 (49.5) |
| **66** | Rwanda | 156 (94.0) | 117 (70.5) | 109 (65.7) | 118 (71.1) | 26 (15.7) | 22 (13.3) | 38 (22.9) | 70 (42.2) | 95 (57.2) |
| **67** | Rwanda | 276 (92.0) | 179 (59.7) | 98 (32.7) | 154 (51.3) | 75 (25.0) | 71 (23.7) | 16 (5.3) | 230 (76.7) | 69 (23.0) |
| **68** | Rwanda | 25 (96.2) | 12 (46.2) | 11 (42.3) | 15 (57.7) | 5 (19.2) | 6 (23.1) | 0 (0.0) | 18 (69.2) | 8 (30.8) |
| **69** | South Africa | 200 (100.0) | 148 (74.0) | 103 (51.5) | 163 (81.5) | 30 (15.0) | 7 (3.5) | 25 (12.5) | 100 (50.0) | 100 (50.0) |
| **70** | South Africa | 220 (100.0) | 50 (22.7) | 6 (2.7) | 93 (42.3) | 117 (53.2) | 10 (4.5) | 7 (3.2) | 202 (91.8) | 12 (5.5) |

**Supplement table 3.** Refusal of consent for outcome assessment *(patient-level analysis)*

|  |  | **Yes refused (N ,%)** | **No did not refuse (N ,%)** |
| --- | --- | --- | --- |
|  | |  |  |
| Randomised allocation | Control | 24 (0.5) | 4825 (99.5) |
|  | Intervention | 17 (0.4) | 4321 (99.6) |
| Age | <18 | 8 (0.9) | 862 (99.1) |
|  | >=18 | 33 (0.4) | 8284 (99.6) |
| Gender | Male | 18 (0.4) | 3962 (99.6) |
|  | Female | 23 (0.4) | 5184 (99.6) |
| Urgency | Elective | 16 (0.4) | 4423 (99.6) |
|  | Emergency | 25 (0.5) | 4723 (99.5) |
| Contamination | Clean-contaminated | 21 (0.4) | 5871 (99.6) |
|  | Contaminated | 12 (0.6) | 1836 (99.4) |
|  | Dirty | 8 (0.6) | 1439 (99.4) |
| Indication | Malignant disease | 8 (0.5) | 1479 (99.5) |
|  | Benign disease | 33 (0.5) | 7207 (99.5) |
|  | Trauma | 0 (0) | 460 (100) |
| Operative approach | Open – midline | 23 (0.4) | 5575 (99.6) |
|  | Open – non-midline | 17 (0.5) | 3362 (99.5) |

**Supplement table 4.** Mapping strategy domains to Cochrane risk of bias tool

| Strategy | Cochrane Risk of Bias-2 Tool |
| --- | --- |
| Minimum of 4 hospitals randomised per country | To reduce imbalance between groups |
| Hospitals identified units of exposure prior to randomisation. Predicted and actual units of exposure were measured | Bias arising from the randomisation process |
| Randomisation was minimised by hospital and country type | To reduce imbalance between groups |
| Training for intervention delivery given after randomisation | Bias arising from the randomisation process |
| Warm up week to test and train trial processes | Bias due to deviations from intended interventions |
| ChEETAh sticker and aggregate register of eligible patients | Bias arising from the randomisation process |
| Communication and monitoring from central team | Bias due to deviations from intended interventions |
| Low-burden post-discharge follow-up | Bias due to missing outcome data |

**Appendix A. Author list**

*Writing group (alphabetical by surname)*

Adesoji O Ademuyiwa, Adewale O. Adisa, Aneel Bhangu, Peter Brocklehurst, Sohini Chakrabortee, Dhruva Ghosh, James C Glasbey, Parvez D Haque, Pollyanna Hardy, Ewen Harrison, JC Allen Ingabire, Lawani Ismail, Bryar Kadir, Rachel Lillywhite, Laura Magill, Antonio Ramos de la Medina, Rachel Moore, Mark Monahan, Dion Morton, Dmitri Nepogodiev, Faustin Ntirenganya, Omar Omar, Thomas Pinkney, Natalie Rowland, Donna Smith, Stephen Tabiri, Neil Winkles

**Collaborators (*denotes the hospital Principle Investigator):**

**Benin**

**Centre Hosptalier Departemental de l’Atacora:** Didier Ahogni, Aristide Ahounou, K. Alassan Boukari, Oswald Gbehade, Thierry K Hessou, Sinama Nindopa, M.J. Bienvenue Nontonwanou, Nafissatou Orou Guessou, Arouna Sambo, Sorekou Victoire Tchati, Affisatou Tchogo, Semevo Romaric Tobome*, Parfait Yanto

**Centre Hospitalier Universitaire et Departemental du Borgou et Alibori:** Isidore Gandaho, Armel Hadonou, Simplice Hinvo, Montcho Adrien Hodonou*, Sambo Bio Tamou

**Centre Hospitalier Universitaire et Departemental Oueme-Plateau:** Souliath Lawani, Covalic Melic Bokossa Kandokponou, Francis Moise Dossou, Antoine Gaou, Roland Goudou, Marie-Claire Kouroumta, Ismail Lawani*, Enrif Malade, Anne stredy Mkoh Dikao, Joel Nzuwa Nsilu, Pencome Ogouyemi

**Centre Hospitalier Universitaire de Zone de Suru Lere:** Marcelin Akpla, Nathan Bisimwa Mitima, Blaise Kovohouande, Cyrille Kpangon*, Stephane Laurent Loupeda

**Hopital Bethesda:** Mamonde Victorin Agbangla, Sena Emmanuel Hedefoun, Thierry Mavoha*, Juvenal Ngaguene, Janvier Rugendabanga, Rish Romaric Soton, Martin Totin

**Hopital de Zone de Dassa-Zoume:** Mouhamed Agbadebo*, Irene Akpo, Hubert Dewamon, Martin Djeto, Aissatou Hada, Monsede Hollo, Albert Houndji, Anasthasie Houndote, Sylvestre Hounsa, Expedit Kpatchassou, Hugues Yome,

**Hopital de Zone de Kandi:** Mohamed Moussa Alidou, Eric Jerry Bara*, B.T. Bonheur Dossou Yovo, Robert Guinnou, Souleymane Hamadou, H.Pauline Kola, Nabil Moussa

**Hopital de Zone de Klouekanme:** Boniface Cakpo, Lolyta Etchisse, Emery Hatangimana, Moise Muhindo, Katia Sanni, Agossou Barthelemy Yevide*

**Hopital de Zone de Menontin:** Hermann Agossou, Fiston Basirwa Musengo, Hulrich Behanzin*, Djifid Morel Seto

**Hopital de Zone de OUIDAH:** Bill Armstrong Alia*, Arnaud Alitonou, Y.Edith Mehounou

**Hopital d’Instruction des Armees de Parakou:** Lucien Agbanda, Julien Attinon, Marcel Gbassi, Nounagnon Rene Hounsou*

**Ghana**

**Berekum Holy Family Hospital:** Regina Acquah, Charles Banka, Derick Esssien, Romeo Hussey*, Yakubu Mustapha, Kojo Nunoo-Ghartey, Grace Yeboah

**Cape Coast Teaching Hospital:** Luke A Aniakwo, Margarey N M Adjei, Yvonne Adofo-Asamoah, Meshach M Agyapong, Thomas Agyen, Baba A B Alhassan, Mabel P Amoako-Boateng, Anthony Baffour Appiah, Josephine Ashong, Joseph K Awindaogo, Benjamin B Brimpong, Makafui S C J K Dayie, Donald Enti, Wendy W Ghansah, Jude E Gyamfi, Patience Koggoh, Richard Kpankpari, Vincent Kudoh, Samuel Mensah, Philip Mensah, Isabella N Morkor Opandoh, Martin T Morna*, Michael Nortey, Emelia Odame, Emmanuel O Ofori, Sandra Quaicoo, Elizabert M Quartson, Cynthia Teye-Topey, Makafui Yigah, Safia Yussif

**Eastern Regional Hospital:** Esther Adjei-Acquah, Vera O Agyekum-Gyimah, Eric Agyemang, Arko Akoto-Ampaw, Forster Amponsah-Manu*, Temitope E Arkorful, Moses A Dokurugu, Nanabanyin Essel, Aja Ijeoma, Emmanuel L Obiri, Richard Ofosu-Akromah, Karen N D Quarchey

**Effia-Nkwanta Regional Hospital:** Leslie Adam-Zakariah*, Aaron B Andoh, Esther Asabre, Ruby A Boateng, Barbara Koomson, Atta Kusiwaa, Adeline Naah, Ato Oppon-Acquah, Benjamin A Oppong

**Greater Accra Regional Hospital:** Emma A Agbowada, Ameley Akosua, Ralph Armah*, Christopher Asare, Lawrence K B Awere-Kyere, Amanda Bruce-Adjei, Nana Ama Christian, Delali A Gakpetor, Korankye K Kennedy, Jacqueline Mends-Odro, Ambe Obbeng, Doris Ofosuhene, Dorcas Osei-Poku, Zelda Robertson

**Komfo Anokye Teaching Hospital:** Dorcas O Acheampong, Jane Acquaye, Juliana Appiah, Joshua Arthur, Jonathan Boakye-Yiadom, Anita Eseenam Agbeko, Frank E Gyamfi*, Bertina B Nyadu

**Korle Bu Teaching Hospital:** Samira Abdulai, Nii A Adu-Aryee, Nelson Agboadoh, Erica Akoto, Joachim K Amoako, Nicholas T Aperkor, Wilfred K Asman, Godsway S Attepor, Antoinette A Bediako-Bowan*, Kwaku Boakye-Yiadom, George D Brown, Florence Dedey, Victor K Etwire, Benjamin S Fenu, Philemon K Kumassah, Linda A Larbi-Siaw, Josephine Nsaful, David O Olatola, Sandra E Tsatsu, Theodore Wordui

**Salaga Municipal Hospital:** Iddrisu I A Abdul-Aziz, Fatao Abubakari, Johnson Akunyam, Gilbert A G Anasara, Cletus Ballu, Charles G Barimah, Guy C Boateng, Ponala W Kwabena, Seidu M Kwarteng, Prosper T Luri*, Kennedy Ngaaso, David K D Ogudi

**Sunyani Regional Hospital:** Vivian Adobea, Amos Bennin, Stanley Doe, Ruth Sarfo Kantanka, Ephraim Kobby, Collins Kyeremeh, Edwin Osei, Prince Yeboah Owusu, Frank Owusu*, Clement Sie-Broni, Marshall Zume

**Tamale Teaching Hospital (Hub):** Saba Abdul-Hafiz, Daniel K Acquah, Shamsudeen M Adams, Mohammed S Alhassan, Munira Amadu, Samuel A Asirifi, Martin Awe, Millicent Azanlerigu, Mathias K Dery, Yenli Edwin, Abantanga Atindaana Francis, Gbana Limann, Aloysius Maalekuu, Hawa Malechi, Sheriff Mohammed*, Ibrahim Mohammed, Kareem Mumuni, Bernard A Ofori, Jonathan I K Quansah, Anwar S Seidu, Stephen Tabiri*, Shekira Yahaya

**Techiman Holy Family Hospital:** Emmanuel Kojo Acquah**,** Jaabir Alhassan, Percy Boakye, Christian L Coompson*, Addo K Gyambibi, Ametepe Jeffery-Felix, Bismark E Kontor, Ruth Manu, Elijah Mensah, Gifty Naah, Carmen Noufuentes, Abraham Sakyi

**India**

**All India Institute of Medical Science, Jodhpur:** Ramkaran Chaudhary, Sanjeev Misra*, Puneet Pareek, Manish Pathak, Dharma R Poonia, Kirti K Rathod, Mahaveer S Rodha, Naveen Sharma, Nivedita Sharma, Subhash C Soni, Vaibhav K Varsheney, Jeevan R Vishnoi

**All India Institute of Medical Science, Rishikesh:** Deepak K Garnaik, Farhanul Huda, Manoj J Lokavarapu, Neha Mishra, Rohit Ranjan, Rajkumar K Seenivasagam*, Shanky Singh, Pratik Solanki, Raunak Verma, Enono Yhoshu

**Baptist Hospital Tezpur:** Suzan John, Jeffery A Kalyanapu, Ananta Kutma, Sanish Philips*

**Christian Hospital Chinchpada:** Arun K Gautham, Alice Hepzibah, Grace Mary, Deepak S Singh*

**Christian Hospital Madhipura:** Eunice S Abraham, Chetana Chetana, Amos Dasari, Prashant Dummala, Chinta S Gold, Jurgen Jacob, Jeremiah N Joseph, Elizabeth N Kurien, Priya Mary, Arpit J Mathew*, Amy E Mathew, Danita D Prakash, Oliver Samuel, Ashwin Sukumar, Niyah Syam, Rose Varghese

**Christian Medical College & Hospital, Ludhiana (Hub):** Alisha Bhatt, William Bhatti, Tapasya Dhar, Dhruva N Ghosh, Ankush Goyal, Sunita Goyal, Monika A Hans, Parvez D Haque, Deepak Jain, Rita Jain, Jyoti Jyoti, Savleen Kaur, Karan Kumar, Anil Luther*, Amit Mahajan, Kavita Mandrelle, Vishal Michael, Partho Mukherjee, Reuben Rajappa, Vivin Daniel Sam, Prashant Singh, Atul Suroy, Ravinder Singh Thind, Sreejith K Veetil, Rahul Williams

**Christian Medical College & Hospital, Vellore:** Sreekar D, Esther R Daniel, Smitha E Jacob, Mark R Jesudason, Pushplatha Kumari, Rohin Mittal*, Soosan Prasad, Vasanth Mark Samuel, Bharat Shankar, Srujan Sharma, Moonish V Sivakumar, Suraj Surendran, Anita Thomas, Paul Trinity

**GB Pant Institute of Postgraduate Medical Education and Research:** Sudheer Kanchodu, K Leshiini, Sundeep S Saluja*

**GMC Chandigargh:** Ashok K Attri*, Ishan Bansal, Sanjay Gupta, Monika Gureh, Simran Kapoor

**GMC Patiala:** Manisha Aggarwal, Vinoth Kanna, Harmanjot Kaur, Ashwani Kumar*, Simrandeep Singh, Gurtaj Singh

**Herbertpur Christian Hospital:** Viju John*

**Kalpana Chawla GMC:** Mohammed Adnan, Nivesh Agrawal, Uttkarsh Kumar, Pardeep Kumar, Abhishek S, Vikram Sehrawat, Deepak Singla, Gaurav Thami*

**Kasturba Medcial College Hospital, Manipal:** Vijay Kumar*, Stanley Mathew, Murlidhar V Pai, P S Prabhu, Sundeep P T

**King George’s Medical Unit:** Naseem Akhtar, Arun Chaturvedi, Sameer Gupta, Vijay Kumar, Puneet Prakash, Shiv Rajan*, Mohit Singh, Abhilasha Tripathi

**Lady Willingdon Hospital, Manali:** Philip V Alexander*, Josy Thomas, Pradeep Zechariah

**Makunda Christian Leprosy & General Hospital:** Vijay A Ismavel, Moloti Kichu*, Carolin V Solomi

**Padhar Hospital:** Rahul A Alpheus, Ashish Victor Choudhrie*, Rashmi Jacob Gunny, Susan Joseph

**PGI Chandigargh:** Muneer A Malik, Nitin J Peters*, Neha Pundir, Ram Samujh

**Sher-I Kashmir Institute of Medical Sciences:** Hafsa I Ahmed, Gowhar Aziz, Nisar A Chowdri, Rayees A Dar, Robindera Kour, Imtiyaz Mantoo, Asif Mehraj*, Fazl Q Parray, Najmus Saqib, Zamir A Shah, Rauf A Wani

**St Stephens Hospital:** Subrat Raul*, Komal Rautela, Rajeev Sharma, Nishu Singh, Rakesh Vakil

**Tata Medical College, Kolkata:** Priyanka Chowdhury, Sona Chowdhury, Sonia Mathai, Pragyanmai Nayak, Bipradas Roy*

**Mexico**

**Centro Medico Nacional de Occidente:** Andrea S Alvarez Villaseñor, Kriscia V Ascencio Díaz, Victor J Avalos Herrera, Francisco J Barbosa Camacho, Aldo Bernal Hernández, Elyoenai Bonilla Ahumada, Irma V Brancaccio Pérez, Miguel A Calderón Llamas, Guadalupe Castillo Cardiel, Guillermo A Cervantes Cardona, Gabino Cervantes Guevara, Enrique Cervantes Perez, Maria Chávez, Jonathan M Chejfec Ciociano, Luis R Cifuentes Andrade, Ana O Cortés Flores, Edgar J Cortes Torres, Tania A Cueto Valadez, Andrea E Cueto Valadez, Esteban Cueva Martinez, Paulina Domínguez Barradas, Isaac Esparza Estrada, Paola Flores Becerril, Jose A Flores Cardoza, Clotilde Fuentes Orozco, Luis A García González, Benjamín García Reyna, Eduardo Gómez Sánchez, Jaime L González Bojorquez, Eduardo González Espinoza, Alejandro González Ojeda*, Fanny Y González Ponce, Cristhian S Guerrero Ramírez, José A Guzmán Barba, Bertha G Guzmán Ramírez, Mario J Guzmán Ruvalcaba, Daniel A Hérnandez Alva, Silvia A Ibarra Camargo, Juan C Ibarrola Peña, Martin Islas Torres, Jorge Jiménez Tornero, Zayra M Lara Pérez, Roberto Mares País, Mel P Mellado Tellez, Roberto C Miranda Ackerman, Damián Mora Santana, Gilberto Morgan Villela, Rodrigo Nájar Hinojosa, Cesar Nuño Escobar, Itzel Ochoa Rodríguez, Oscar Olvera Flores, Angelica Ortega Barreiro, Jacqueline Osuna Rubio, Luis R Pacheco Vallejo, Víctor H Pérez Bocanegra, Jose V Pérez Navarro, Francisco J Plascencia Posada, María A Quirarte Hernández, Luis R Ramirez Gonzalez, Emilio A Reyes Elizalde, Evelia V Romo Ascencio, Cornelio Ruelas Bravo, Carlos B Ruiz Velasco, José A Sánchez Martínez, Guillermo Sanchez Villaseñor, José I Sandoval Pulido, Alejandro G Serrano García, Luis O Suárez Carreón, Juan J Tijerina Ávila, Jesus O Vega Gastelum, Melissa L Vicencio Ramirez, Maria F Zarate Casas, Carlos J Zuloaga Fernández del Valle

**Hospital Civil de Guadalajara:** Jesus Antonion Aguilar Mata, Miguel Antonio Calderon Vanegas, Rocio Guadalupe Cano Arias, Carlos Colunga Tinajero, Fernanda Diaz Samano, Fernando Duque Zepeda, Brenda Vanessa Enriquez Barajas, Gerardo Gallardo Banuelos, Marijose De Cristo Gonzalez Calvillo, Francisco Ibanez Ortiz, Maryzela Lazo Ramirez, Gerardo Lopez Arroyo, Laura Olivia Montano Angeles, David Giovanny I Morales Iriarte, Angelo Fernando Mortola Lomeli, Jose Esteban Orozco Navarro, Jaime Orozco Perez, Damaris Orozco Ramirez, Laura Gabriela Pena Baolboa, Jesus Pizarro Lozano, Guillermo Yanowsky Reyes*

**Hospital De Especialidades Del Nino Y La Mujer:** Monica N Castillo*, Ana Camille G Dominguez, Dorihela H Mellado, Jesus Flavio M Morales, Luz del Carmen M Namur, Jose Alberto A Pesquera

**Hospital Espanol Veracruz (Hub):** Laura Martinez Perez Maldonado, Antonio Ramos De la Medina

**Hospital General Dr. Manuel Gea Gonzalez:** Katya Bozada-Gutierrez, Ana Florencia Casado-Zarate, Roberto Delano-Alonso, Jose Herrera-Esquivel, Mucio Moreno-Portillo, Mario Trejo-Avila*

**Hospital Regional de Alta Especialidad del Bajio:** Roland Kevin Cethorth Fonseca, Edgard Efren Lozada Hernandez*, Bruno Crocco Quiros, Jairo Arturo Rodriguez Ramirez

**UMAE Hospital de Pediatria CMNO:** Gabriela Ambriz-González*, Mitzi R Becerra Moscoso, Ishtar Cabrera-Lozano, Ana B Calderón-Alvarado, Francisco J León-Frutos, Erick E Villanueva-Martínez

**Nigeria**

**Ahmadu Bello University Teaching Hospital:**Aisha Abdullahi, Maimuna Abubakar, Mohammed S Aliyu, Mudi Awaisu, Fadimatu Bakari, Abigail Olajumoke Balogun, Mohammed Bashir, Ahmad Bello, Muhammad Daniyan, Kehinde Michael Duromola, Stephen G Gana, Mukoro Duke George, Justina Gimba, Isaac Gundu, Lambert Onahi Iji, Aminat O Jimoh, Afolabi K Koledade, Ahmad T Lawal, Bilkisu K Lawal, Aisha Mustapha, Stanley Emeka Nwabuoku, Oluseyi O Ogunsua, Ifeanyi Fidelis Okafor, Ethos Ike Okorie, Nasir Oyelowo, Ibrahim A Saidu, Tunde T Sholadoye, Ibrahim Sufyan, Musliu Adetola Tolani*, Aliyu Muhammad Tukur, Ahmad Shehu Umar, Aminatu M Umar, Hajara Umaru-Sule, Mohammed Usman, Anisah Yahya, Alfa Yakubu, Salisu Abeku Yusuf

**Aminu Kano Teaching Hospital:** Abdulhafiz A Abdulkarim, Lawal Barau Abdullahi, Muzzammil Abdullahi, Khadija A Ado, Nura U Aliyu, Lofty-John Chukwuemeka  Anyanwu*, Sulaiman M Daneji, Mahmoud Kawu Magashi, Mohammad A Mohammad, Abubakar Bala Muhammad, Saminu S Muhammad, Bello Abodunde Muideen, Calistus U Nwachukwu, Suleiman B Sallau, Abdulrahman A Sheshe, Abdulmajeed Soladoye, Idris Usman Takai, Garzali I Umar, Abubakar Yahaya

**Barau Dikko Teaching Hospital:**Lubabatu Abdulrasheed, Joel A Adze, Lydia R Airede, Bashiru Aminu, Stephen B Bature, Firdaws Bello-Tukur, Damai Chinyio, Sharon A N Duniya, Moses C Galadima, Babatunde K Hamza, Samaila Joshua, Stephen A Kache*, Williams Y Kagomi, Ifeanyi A Kene, Jamila Lawal, Jerry G Makama, Caleb Mohammed, Amina A Mohammed-Durosinlorun, Deborah Nuwam, Danjuma Sale, Abdulrasheed Sani, Salome Tabara, Mathew C Taingson, Emmanuel Usam, Josiah Yakubu

**Federal Medical Centre, Owo:**Folasade Adegoke, Oluwasuyi Ige, Tunde A Odunafolabi, Chukwuma E Okereke*, Oluwafemi O Oladele, Oluwaseun H Olaleye, Oyetunde O Olubayo

**Federal Teaching Hospital, Ido-Ekiti:**Olukayode P Abiola, Henry O Abiyere, Idowu O Adebara, Gbadebo T C Adeleye, Adebayo A Adeniyi, Olumide E Adewara, Olabisi T Adeyemo, Ademola A Adeyeye, Abimbola L Ariyibi, Babatunde S Awoyinka, Olumide M Ayankunle, Olakunle F Babalola, Adewumi Bakare, Tajudeen I B Bakare, Oluseyi O Banjo, Peter A Egharevba, Oluwafemi S Fatudimu, John A Obateru, Oluremi J Odesanya, Owolabi D Ojo, Abiodun I Okunlola*, Cecilia K Okunlola, Adewale T Olajide, Tesleem O Orewole, Adedayo I Salawu

**Lagos State University Teaching Hospital:**Moruf A Abdulsalam, Aderinsola T Adelaja, Olalekan T Ajai, Olukemi Akande, Noble Anyanwu, Kazeem M Atobatele, Oludayo Oluwaseyi Bakare, Grace Eke, Omolara M Faboya, Zainab O Imam, Francisca C Nwaenyi, Ayokunle A Ogunyemi, Mobolaji A Oludara, Olufunmilade A Omisanjo, Chinonso U Onyeka, Olabode A Oshodi, Yusuf A Oshodi, Yemisi Oyewole, Omotade S Salami, Omolara M Williams*

**Lagos University Teaching Hospital:**Esther Abunimye, Adesoji O Ademuyiwa*, Adebunmi Adeoluwa, Adedotun Adesiyakan, Victoria Ibukunoluwa Adeyeye, Moses Vincent Agbulu, Opeyemi Rebecca Akinajo, David O Akinboyewa, Felix M Alakaloko, Iyabo O Alasi, Michael Amao, Christiana Ashley-Osuzoka, Oluwole A Atoyebi, Olanrewaju S Balogun, Christopher O Bode, Maryam Oluwatobi Busari, Nnamdi Jonathan Duru, Glory Bassey Edet, Olumide A Elebute, Francis Chinonso Ezenwankwo, Adedeji L Fatuga, Christianah Gbenga-Oke, George C Ihediwa, Emmanuel Sylvester Inyang, Adesola I Jimoh, Jubril Oladayo Kuku, Oluwaseun A Ladipo-Ajayi, Abdulrazzaq O Lawal, Ayomide Makanjuola, Christian Chigoze Makwe, Chinelo Victoria Mgbemena, Samuel U Nwokocha, Moses Adebisi Ogunjimi, Ephraim Okwudiri Ohazurike, Rufus W Ojewola, Moyosoluwa Eunice Badedale, Chike J Okeke, Adeyemi A Okunowo, Abraham T Oladimeji, Thomas O Olajide, Olabisi Olanrewaju, Olawunmi Olayioye, Oluwaseun O Oluseye, Stephen Olutola, Kenneth Onyekachi, Adeola Ayoola Orowale, Emili Osariemen, Adedapo Olumide Osinowo, Benedetto Osunwusi, Emmanuel Owie, Christianah Bidemi Oyegbola, Justina O Seyi-Olajide, Adaiah P Soibi-Harry, Manuella Talla Timo, Aloy Okechukwu Ugwu, Emmanuel Ojo Williams

**Nnamdi Azikiwe University Teaching Hospital:**Innocent O Duruewuru, Ochonma A Egwuonwu, Okechukwu Hyginus Ekwunife*, James J Emeka, Victor Ifeanyichukwu Modekwe, Chimdiebele Daisy Nwosu, Sylvester O Obiechina, Ahuizechukwu E Obiesie, Celestine I Okafor, Theophilus O Okonoboh, Chukwuemeka Okoro, Odili A Okoye, Onyekachi A Onu, Chukwudubem C Onyejiaka, Chisom Faith Uche, Joseph O Ugboajah, Jideofor Okechukwu Ugwu, Kenneth Ugwuanyi, Chuka Ugwunne

**Obafemi Awolowo University Teaching Hospitals Complex, Ile-Ife:**Akeem A Adeleke, Akinfolarin C Adepiti, Adewale A Aderounmu, Abdulhafiz O Adesunkanmi, Adewale O Adisa*, Samuel C Ajekwu, Olusegun K Ajenifuja, Olusegun I Alatise, Tajudeen A Badmus, Tajudeen O Mohammed, Olalekan Olasehinde, Abdulkadir A Salako, Oludayo A Sowande, Ademola O Talabi, Funmilola O Wuraola

**O.L.A. Catholic Hospital, Oluyoro, Ibadan:**Paul Aderemi Adegoke, Abidemi Akinloye, Ayodeji Akinniyi, Joseph Ejimogu, Ideyonbe Samuel Eseile, Olakayode Olaolu Ogundoyin*, Amos Okedare, Dare Isaac Olulana, Omolara Omotola, Francis Sanwo

**Uniosun Teaching Hospital:**Collins C Adumah, Adewale O Ajagbe, Olugbenga P Akintunde, Opeyemi Q Asafa, Kehinde Awodele, Amogu K Eziyi, Adeniyi O Fasanu, Olufemi O Ojewuyi*, Abiodun R Ojewuyi, Abisola E Oyedele, Oluwaseun A Taiwo

**University of Abuja Teaching Hospital:**Habiba I Abdullahi, Nathaniel D Adewole, Teddy E Agida, Eunice E Ailunia, Oseremen Aisuodionoe-Shadrach, Godwin O Akaba, Janet Alfred, Terkaa Atim, Kehinde G Bawa, John Y Chinda, Esther B Daluk, Sefiu B Eniola, Augusta O Ezenwa, Stephen E Garba, Ndubuisi Mbajiekwe, Philip M Mshelbwala, Ngozi O Ndukwe, Idoko P Ogolekwu, Alexander A Ohemu, Samson Olori*, Olabisi O Osagie, Samuel A Sani, Salisu Suleiman, Helen Sunday, Nancy O Tabuanu, Aminu M Umar

**University of Benin Teaching Hospital:**Peter I Agbonrofo*, Alexander I Arekhandia, Morrison E Edena, Raymond A Eghonghon, Joel E Enaholo, Genesis Ida, Stanley N Ideh, Oseihie I Iribhogbe, Omorodion O Irowa, Maradona E Isikhuemen, Oluwatomi R Odutola, Kester O Okoduwa, Scott O Omorogbe, David Oruade, Osasumwen T Osagie, Osarenkhoe Osemwegie

**University College Hospital, Ibadan:**Rukiyat A Abdus-Salam, Sikiru Adekola Adebayo, Oluwasanmi A Ajagbe, Akinlabi E Ajao, Gboyega Ajibola, Omobolaji O Ayandipo, Kelvin I Egbuchulem, Hyginus O Ekwuazi, Peter Elemile, Adegbolahan Fakoya, Oluwasegun C Idowu, David O Irabor, Taiwo A Lawal*, Olatunji O Lawal, Olakayode O Ogundoyin, Oluwabukade Ojediran, Naomi Olagunju, Akinsola T Sanusi, Augustine O Takure

**University of Ilorin Teaching Hospital:** Lukman Olajide Abdur-Rahman*, Mary Oluwadamilola Adebisi, Nurudeen Abiola Adeleke, Rafiat Tinuola Afolabi, Isiaka Ishola Aremu, Jibril Oyekunle Bello, Robiat Bello, Abdulwahab Lawal, Saheed Abolade Lawal, Adeolu Ojajuni, Sabur Oyewale, Hadijat Olaide Raji, Olayinka Sayomi, Asimiyu Shittu

**University of Port Harcourt Teaching Hospital:**Victor Abhulimen, Patrick O Igwe, Ikechukwu Enyinnaya Iweha, Raphael E John, Nnyonno Okoi, Philemon E Okoro*, Vaduneme Kingsley Oriji, Ibiene T Oweredaba

**Rwanda**

**Bushenge Provincial Hospital:** Japhet Mizero, Immaculee Mutimamwiza, Francoise Nirere, Irenee Niyongombwa*

**Butaro Hospital:** Jean Paul Majyabere*

**Byumba Hospital:** Anastase Byaruhanga, Rongin Dukuzimana, Jean Aimable Habiyakare*, Marie Gloriose Nabada, Marcel Uwizeye

**Kabgayi Hospital:** Mathias Ruhosha*

**Kibagabaga District Hospital:** joselyne Igiraneza, Faustine Ingabire, Aloys Karekezi, Jean pierre Masengesho, Christophe Mpirimbanyi*, Lydia Mukamazera, Clemence Mukangabo, Jean Paul Niyomuremyi, Gabriel Ntwari, Celestin Seneza, Divine Umuhoza

**Kibogora Hospital:** Sosthene Habumuremyi, Alphonsine Imanishimwe, Salathiel Kanyarukiko, Francine Mukaneza, Deborah Mukantibaziyaremye, Aphrodis Munyaneza, Gibert Ndegamiye, Pierrine Nyirangeri, Ronald Tubasiime*, Jean Claude Uwimana

**Kibungo Referral Hospital:** Moses Dusabe, Emelyne Izabiriza, Hope Lydia Maniraguha, Christophe Mpirimbanyi*, Josiane Mutuyimana, Olivier Mwenedata, Elisee Rwagahirima, Job Zirikana

**Kibuye Referral Hospital:** Isaie Sibomana*

**King Faisal Hospital:** Desire Rubanguka*, Josine Umuhoza, Roda Uwayezu, Leoncie Uzikwambara

**Ruhengeri Referral Hospital:** Aime Dieudonne Hirwa*, Elysee Kabanda, Salomee Mbonimpaye, Christine Mukakomite, Piolette Muroruhirwe

**Rwamagana Provincial Hospital:** Herbert Butana*, Moise Dusabeyezu, Athanasie Mukasine, Jean N Utumatwishima

**Rwanda University Teaching Hospital of Kigali (Hub):** Mediatrice Batangana, Georges Bucyibaruta, Sosthene Habumuremyi, Jean de Dieu Haragirimana, Alphonsine Imanishimwe, Allen J C Ingabire, Violette Mukanyange, Emmanuel Munyaneza, Emmanuel Mutabazi*, Espoir Mwungura, Isaie Ncogoza, Faustin Ntirenganya*, Jeannette Nyirahabimana, Dancilla Nyirasebura, Christian Jean Urimubabo

**University Hospital of Butare:** Anaclet Dusabimana, Sam Kanyesigye, Robert Munyaneza*, Jean Yves Shyirakera

**South Africa**

**Chris Hani Baragwaneth (Hub):** Maria Fourtounas, Mary Augusta Adams, Chikwendu Jeffrey Ede, Gabriella Hyman, Mpho Nosipho Mathe, Rachel Moore*, Ncamsile Anthea Nhlabathi, Hlengiwe Samkelisiwe Nxumalo, Nnosa Sentholang, Mmule Evelyn Sethoana, Paul Wondoh

**Helen Joseph Hospital**: Zain Ally*, Aimee Domingo, Philip Munda, Chido Nyatsambo, Victor Ojo, Rudo Pswarayi

**Appendix B: Strategies following identification of potential sources of bias**

A number of strategies were deployed following identification of potential sources of bias and concerns around adherence rates.

Routine central monitoring of the CHEETAH aggregate registers data (to monitor the total number of patients eligible for the trial), versus the *actual* patients registered on the REDCap trial database allowed the central trials team to quickly identify sites where some bias had potentially occurred, in particular one site was identified which facilitated further investigation. Swift action involved a teleconference call between the CI/central trials team and the in-country Hub team to (a) quickly identify the potential areas of concern, (b) allow the central trials team to define and share some preventative measures particularly around the patient pathway processes and (c) determine a strategy to minimise further issues which included reducing the number of pre-identified (*predicted*) emergency CHEETAH theatres to a more manageable number of CHEETAH theatres to ensure consecutive recruitment of ALL abdominal surgical patients.

Lessons learned were immediately shared across the GSU network with the Hub Directors both in writing via email, and directly via teleconference, information was also shared with all Hub Managers for dissemination to their Hub and Spoke teams. Hub Leads communicated the findings via a number of in-country trial-specific meetings and/or via email directly to site leads.

Fundamentally, corrective and preventative measures were largely around re-training and the provision of additional guidance to ALL sites reinforcing a number of key trial processes including; (i) a reminder to sites completion of the operation sticker is mandatory for ALL abdominal cases going through the theatres identified as CHEETAH theatres, (ii) re-emphasising the operation sticker must be an accurate reflection of what *actually* happened in theatre not what *should* have happened according to hospital randomisation (iii) re-iterating ALL pre-defined theatres (prior to site opening) must open to CHEETAH and ALL identified theatres MUST open at the same time and begin consecutive recruitment at the time of site activation, (iv) encouraged sites to make good use of laminated theatre wall charts to visually remind theatre teams of the randomised site allocation and the associated trial processes, (v) re-emphasised the importance of the CHEETAH Registers to be completed each day from the operations listed on the standard theatre logbooks from all participating CHEETAH theatres and must include ALL abdominal surgeries performed in those theatres, (vi) encouraged timely completion of the CHEETAH Aggregate register to aid central monitoring for bias.

As a result of this communication, a few sites confirmed some patients had not been included if for example the intervention of change of gloves/instruments had not been performed. Early identification of discrepancies allowed us to identify sites where bias may have occurred and to correct this by instructing sites to revisit their operation lists and CHEETAH registers and immediately enter any `missed` patients i.e. patients that may have been excluded because the intervention of change of gloves/instruments was not performed as per hospital randomisation. All patients that met the eligibility criteria were retrospectively registered into CHEETAH. Where cases were not included and the site were unable to make contact with the patient these have been recorded and included in the final paper.

Open communication between the central trials team at BCTU, Hub(s) and Spoke(s) along with central monitoring of CHEETAH registers and the CHEETAH trial data uploaded on the REDCap database, was key to managing bias. Any discrepancies were quickly identified, communicated and queried via Hub Managers/Teams until resolution.

**Predicted vs Actual Theatres**

Central monitoring also included monitoring of *predicted* vs *actual* participating CHEETAH theatres any discrepancies between the two, were again queried and explanations sought via in-country Hub teams.

**Monitoring for evidence of bias**

Monitoring for evidence of bias and ensuring consecutive recruitment is critical to the integrity of the data in CHEETAH. The hospital`s standard theatre logbook/register is reviewed during on-site visits or via tele/videoconference if done remotely to confirm all eligible cases have been included, compared with the patients that have been registered in CHEETAH according to the CHEETAH Register, the CHEETAH REDCap database and the CHEETAH Identification Log. Prior to monitoring visits a proportion of CHEETAH registers are selected, at random, by the programming team at BCTU to be reviewed during site monitoring visits/calls.

Monitoring of the sites both centrally and via on-site visits helped to provide further reassurances that adherence (and other trial processes) were being performed and reported correctly. Prior to restrictions around travel the central trials team from the Birmingham Clinical Trials Unit (BCTU) had planned visits to monitor the Hub teams along with a selection of in-country Spoke hospitals where possible. Unfortunately, the COVID pandemic prevented travel and alternative plans were developed to monitor sites remotely via tele/videoconference. A pre-defined monitoring plan, checklist(s) and guidance document set was developed and Hub teams were trained in their use by the central teams at BCTU. In line with our Hub-Spoke model, BCTU monitored Hub sites and trained Hub teams in order for them to monitor and manage oversight of their Spokes in each country.

The purpose of the Monitoring checks *(not limited to):*

- to assess adherence to the trial protocol
- to determine compliance with key CHEETAH pathway processes e.g. completion of the CHEETAH operation sticker, completion of the CHEETAH register(s)
- to ensure that consecutive patients were included, by comparing the standard theatre logbook(s) with the CHEETAH registers
- to determine if the pre-defined (*predicted*) CHEETAH theatres aligns with the *actual* theatres participating and registering patients into the trial

**Appendix C: ChEETAh trial-specific register**


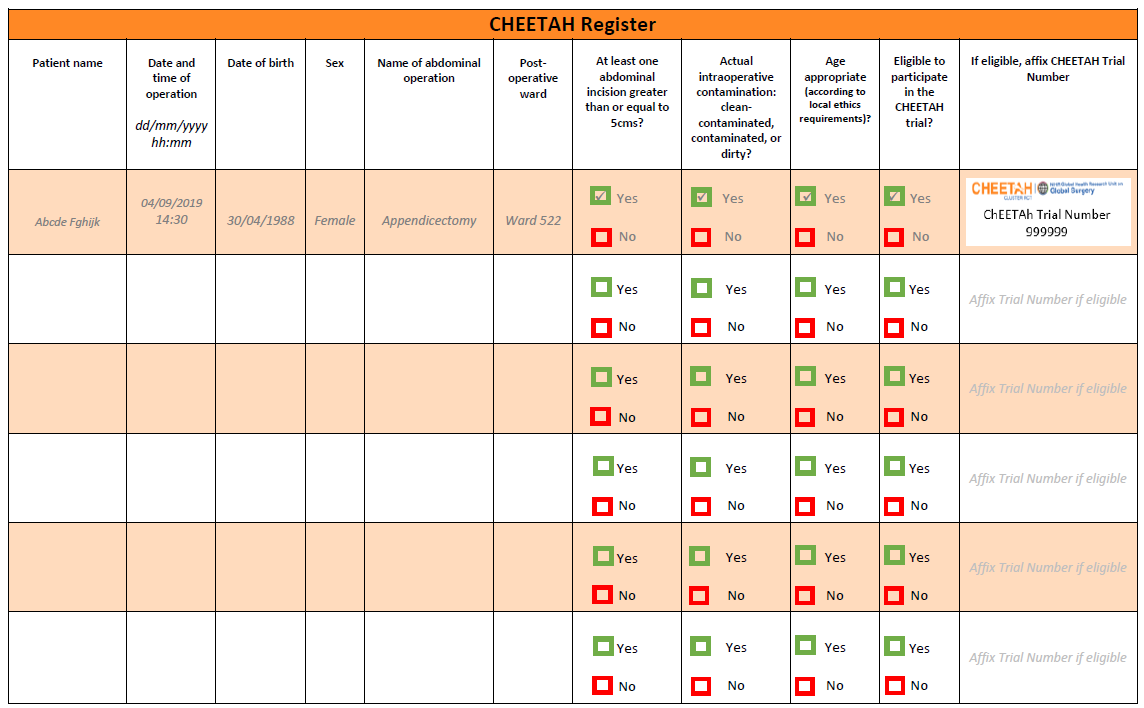


**Appendix D: ChEETAh trial operation sticker**


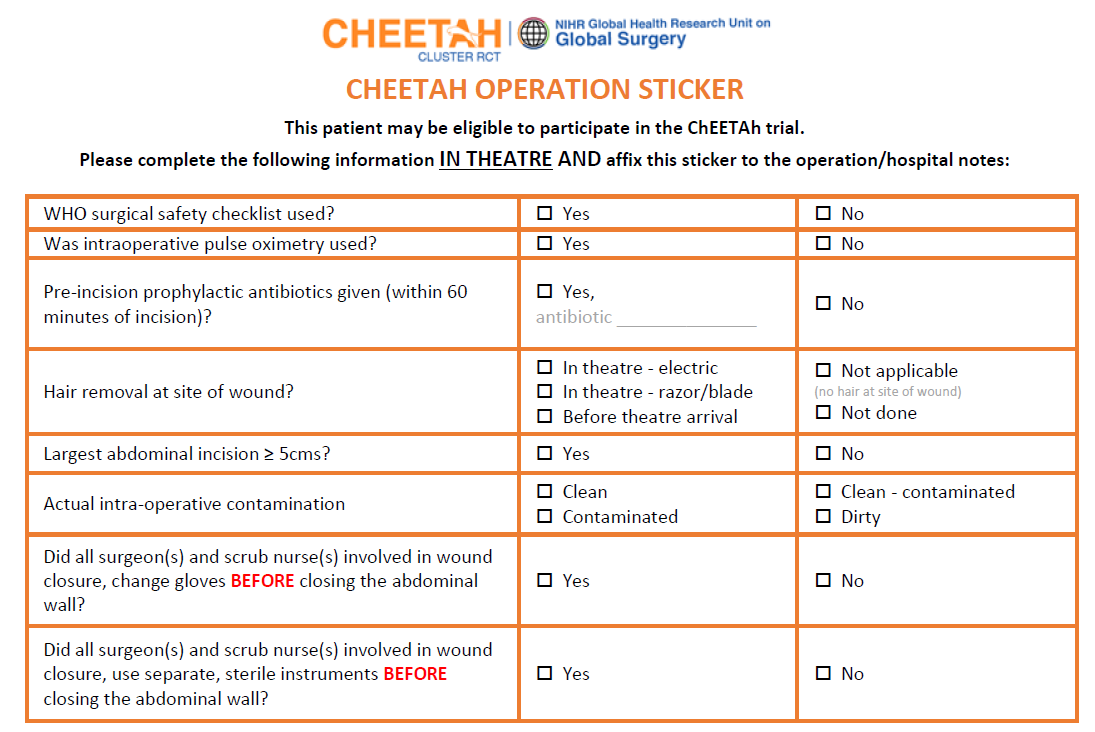


**Appendix E: ChEETAh aggregate register**


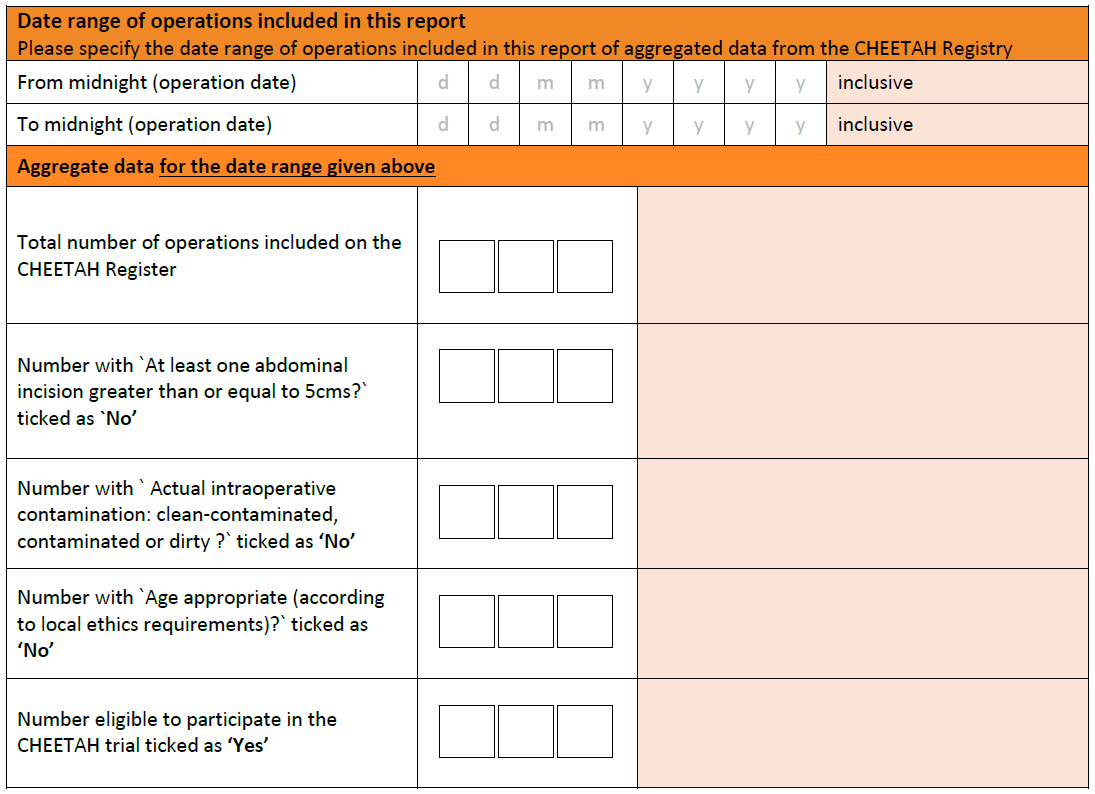


**Appendix F: ChEETAh Patient Pathway Flowchart**


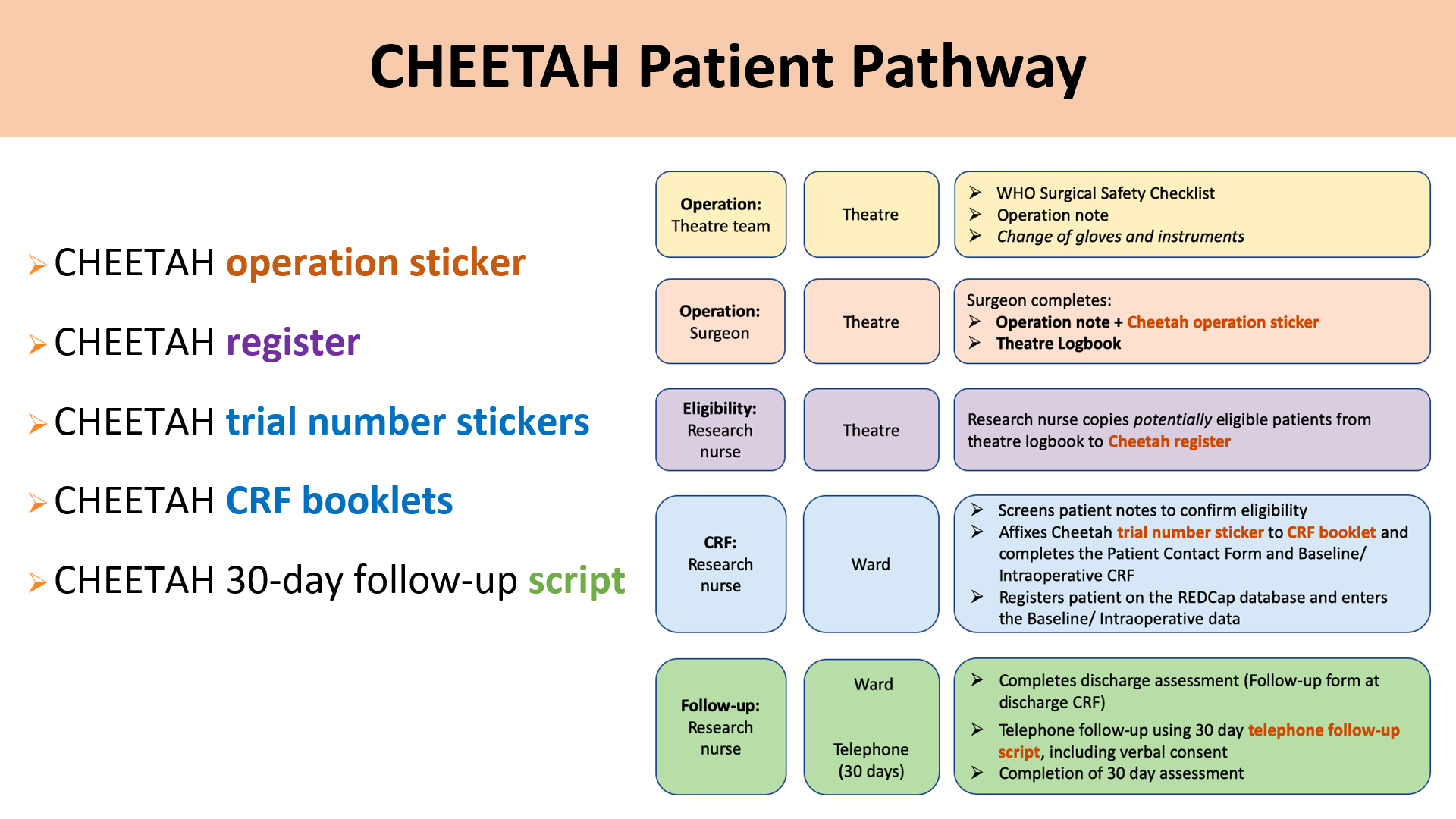

Supplement: Supplementary file 1 — Additional file 1: Fig. S1. Balance of elective and emergency surgery by trial arm. Fig. S2. Changes in imbalance by arm of urgency of surgery over time. Fig. S3. Flowchart of key surgery characteristics overall. Fig. S4. Number of actual and predicted units of exposure per hospital. Table S1. Number of eligible patients included (patient-level analysis). Table S2. Baseline characteristics of included patients (N=, %) (hospital-level analysis). Table S3. Refusal of consent for outcome assessment (patient-level analysis). Table S5. Mapping strategy domains to Cochrane risk of bias tool. Appendix A. Author list. Appendix B. Strategies following identification of potential sources of bias. Appendix C. ChEETAh trial-specific register. Appendix D. ChEETAh trial operation sticker. Appendix E. ChEETAh aggregate register. Appendix F. ChEETAh Patient Pathway Flowchart. [file 13063_2022_6852_MOESM1_ESM.docx]
